# Continuous cropping of endangered therapeutic plants *via* electron beam soil-treatment and neutron tomography

**DOI:** 10.1038/s41598-018-20124-7

**Published:** 2018-02-01

**Authors:** Cheul Muu Sim, Bong Jae Seong, Dong Won Kim, Yong Bum Kim, Seung Gon Wi, Gyuil Kim, Hwasuk Oh, TaeJoo Kim, Byung Yeoup Chung, Jeong Young Song, Hong Gi Kim, Sang-Keun Oh, Young Dol Shin, Jea Hwan Seok, Min Young Kang, Yunhee Lee, Mabuti Jacob Radebe, Nikolay Kardjilov, Bernd Honermeier

**Affiliations:** 1 Korea Atomic Energy Research Institute, 1045 Daedeokdaero Yuseong-gu, Daejeon, 303-353 Korea; 2 Geumsan Ginseng & Medicinal Crop Experiment Station, Chungnam, 312-831 Korea; 3 Specialized Crop Research Institute, Jinan gun, Jeonbuk, 567-807 Korea; 4 National Institute of Horticulture & Herb Science, Bisani 80, Eumseong, Chungbuk, 389-873 Korea; 5 Bioenergy Research Institute, Chonnam National University, 300 Yongbong-dong, Buk-gu, Gwangju, 500-757 Korea; 6 Institute of Jinan Red Ginseng, Jinan gun, Jeonbuk, 567-801 Korea; 7 RIC for Next Generation Industrial Radiation Technology, Wonkwang University. 460, Iksan-daero, Iksan-si, Jeollabuk-do, 54538 Korea; 8 Chungnam National University, 220 Gung-dong, Yuseong-gu, Daejeon, 305-764 Korea; 9 Plant Genomics and Breeding Institutes, Seoul National University, Gwanak-gu, Seoul, 151-921 Korea; 10 GBioMix Institute, 723-1, 2 Palbok-dong, Deokjin-gu, Jeonju, 561-844 Korea; 11 Chonbuk National University, 567 Baekje-daero, Deokjin-gu, Jeonju, 561-756 Korea; 12 King’s College London, Palace Road, London, SE1 7EH UK; 13 Nuclear Energy Corporation South Africa, 0001 Pretoria, South Africa; 14 Helmholtz Zentrum Berlin, 14109 Berlin, Germany; 15 Justus Liebig University Gießen, Schubertstr. 81, D-35392 Gießen, Germany

## Abstract

Various medicinal plants are threatened with extinction owing to their over-exploitation and the prevalence of soil borne pathogens. In this study, soils infected with root-rot pathogens, which prevent continuous-cropping, were treated with an electron beam. The level of soil-borne fungus was reduced to ≤0.01% by soil electron beam treatment without appreciable effects on the levels of antagonistic microorganism or on the physicochemical properties of the soil. The survival rate of 4-year-old plant was higher in electron beam-treated soil (81.0%) than in fumigated (62.5%), virgin (78%), or untreated-replanting soil (0%). Additionally, under various soils conditions, neutron tomography permitted the monitoring of plant health and the detection of root pathological changes over a period of 4–6 years by quantitatively measuring root water content *in situ*. These methods allow continual cropping on the same soil without pesticide treatment. This is a major step toward the environmentally friendly production of endangered therapeutic herbs.

## Introduction

The use of medicinal plants, both in allopathic and herbal medicine, is expected to rise globally^1,2^. This is due to the increasing number of elderly people and to the preference of consumers for natural and eco-friendly products. The increasing use^3^ of natural medications has renewed interest in the extraction of medicinal compounds from roots, which increases pressure on plant resources. The demand for phytotherapeutic roots is soaring; however, the availability of the products has remained limited in soils affected by soil-borne pathogens. Consequently, eco-friendly soil management techniques are needed to conserve and continually produce rare plant species^4–6^. Over-exploitation of wild-medicinal plants and the rampancy of soil-borne pathogens would destroy the habitat of several rarity plant such as *Astragalus*^7^, *Dioscorea*^5^, *Glycyrrhiza*^8^, *Harpagophytums*^9^, *Panax ginseng*^10^, *Rauvolfia*^11^, and *Valeriana*^12^. The species mentioned above are perennial plants with roots from which valuable natural medicines can be produced. The roots of these plants are highly prized because of their unique pharmacological effects they elicit, including immunomodulatory^7^, anti-inflammatory^8^, and antioxidant^10^ effects. These medicines can be used to treat hypertension^9^, cardiovascular disease^11^, insomnia^12^, and HIV^10^. These species are threatened with extinction due to their extreme-utilization and the prevalence of a variety of soil-borne pathogens^4–6^.

Cultivation of these perennial plants in the shade at high temperatures results in high rates of photorespiration and promotes the growth of fungal pathogens. These plant diseases become increasingly apparent during dehiscence, germination, leaf growth, offshoot formation, flowering, and ripening. These plants require at least 2-6 years to mature, even under the most favorable cultivation conditions, and their yields are much lower than those of other crops^13^. This prolonged growth period of endangered phytotherapeutic-root plants makes them highly vulnerable to root infections by soil-borne fungal pathogens such as *Phytophthora* spp., *Rhizoctonia solani*, *Pythium*, *Fusarium solani*, and *Cylindrocarpon* spp.^14^. The cultivation fields cannot be replanted due to the build-up of inocula from a range of plant soil pathogens^15^. Therefore, the root products are in short supply. The sterilization of soil containing pathogens by a method other than chemical pesticide treatments has not been reported before. If farmers want to avoid pesticide usage, they must use virgin soil that has never been planted with roots before, which incurs high economic and environmental costs.

*Cylindrocarpon destructans* (*C*. *destructans*)^16–19^ is the major root-rot pathogen of various plants and trees that affects root, although other factors may be involved^14,20,21^. The foliage is managed by reducing the leaf wetness and applying fungicidal sprays, but root rot is very difficult to control. *C*. *destructans*, a dense parasite that causes decay *via* infection of woody seedling infections of a variety of hosts, produces a thick-walled chlamydospore^14,21^. The pathogen cannot be eliminated completely from fields using a combination of heat, chemicals, high pressure, and filtration^14^.

Nevertheless, pre-seeding fumigation has been widely used for soil-borne disease management, although overdoses of fungicides may promote the development of resistant pathogens and the carryover of harmful chemical residuals into this medicinal crop^22^. The level of *C*. *destructans*, the major root rot pathogen that prevents continuous cropping, was reduced by chemically fumigation treatment with appreciable effects on the levels of Actinomyces, aerobic bacteria, or the physicochemical properties of the soil. Methyl bromide and Dazomet were the most widely used fumigant, but theirs production and usage were restricted because they caused ozone depletion and was toxic to humans^23^. Additionally, a procedure of theirs application is too complex to sterilize soil-borne pathogen and they changes the soil environment and resident microorganisms^24^.

The current solution to these problems is the use of virgin fields for new crops. Agricultural migration for new fields sustains rare species cropping, but the economic and environmental costs are high. Eco-friendly soil management other than in fumigated or virgin soils is indeed inevitable. This study reports an eco-friendly technology that can be used to sterilize cultivation soil. The extinction of many plants used for medicinal purposes could be prevented, and they could be continually cultivated by applying electron beam soil treatment while neutron tomography is used to monitor water content and morphology of roots.

## Electron beam treatment

In 1960, studies showed that EB treatment of soil maintains its microbial population with few physical and chemical changes, whereas chemical and heat treatments have various physical and chemical effects^24–27^. Previous studies revealed that the increased availability of mineral released from the breakdown of organic compounds in the soil by the radiation stimulates plant growth. The study, however, does not determine the mortality of a certain fungi in association with plant pathology. The major applications of EB in association with water radiolysis include grafting, sterilization of food and waste water, and inactivation of hazardous genetic substance^28,29^. EB also is used to eliminate pathogens during the disinfestation of wheat^30^.

The present study demonstrates that electron beam (EB) treatment combined with antagonistic biological-control^31^ can be used as a novel sterilization method to avoid replanting diseases of used cultivation soil for continuous cropping. *Panax ginseng*, an important phytotherapeutic herb plants, was planted for the experiments. It suffers severely from chronic continuous cropping disease, caused by *C*. *destructans* that develops over a 4-6 year cultivation period. EB effects on bacterial and fungal mortality were first investigated in the EB-treated soil when assessing the plant growth of different treatment. The effects of *C*. *destructans*, *Fusarium solani*, *Alternaria solani*, *Actinomyces*, and *Bacillus amyloliquefaciens* were investigated in the EB treated soil. The density of *C*. *destructans* was periodically determined by real-time polymerase chain reaction (PCR)^32,33^. Variations in the physicochemical properties of the irradiated soil were examined. A field test, using earthworms in control and experimental soils, was conducted to confirm the ecological effects of the EB treatment. The crops were grown in irradiated replanting soil (RS) and first planting soil (FS). The emergence rates were observed in fumigated soil, EB-treated soil, untreated soil, and virgin soil. EB soil treatment allows ecologically sustainable cultivation and the continuous harvesting of roots from the same land. Thus, EB soil treatment could replace the use of pesticides and nutrient solutions used in protected cultivation and satisfy the soaring demand for medicinal plants. This novel method is also suited to modern agricultural methods such as greenhouses and plant factories.

### Neutron tomography

Neutron^34^ radiography^35,36^, which images interactions within the nucleus of atoms, rather than between electrons like X-ray^37^, can identify the strongly interacting hydrogen in water molecules, and can be used to determine water movement in soils and plants^38–41^. The first of serial neutron tomography (NT)^42^ was used to determine the water content and morphology of roots planted in pots embedded in the field. The results of serial tomography were used to diagnose root diseases *in situ*.

## Results

### Field trial test formation for EB irradiation soil

The present study conducted the first cultivation experiment to assess the suitability of EB treatment of soil-borne pathogen for crop improvement in two fields. One hundred tons of soil from fields in which roots had already been grown for 6 years were treated at an EB irradiation facility and then used to construct the experimental fields (Supplementary-1 Fig. 4). One field, located in Geumsan, South Korea, was used for a series of experiments (from 1997–2006) that involved fumigation and neutron imaging to diagnose root pathologies. The other field, located in Jinan, South Korea, was used for a series of experiments (from 2007–2012) that involved EB soil treatment and peripheral neutron imaging to measure water level while the roots were embedded in the soil.

### Crop Improvement after EB irradiation of soil

**Figure 1:**
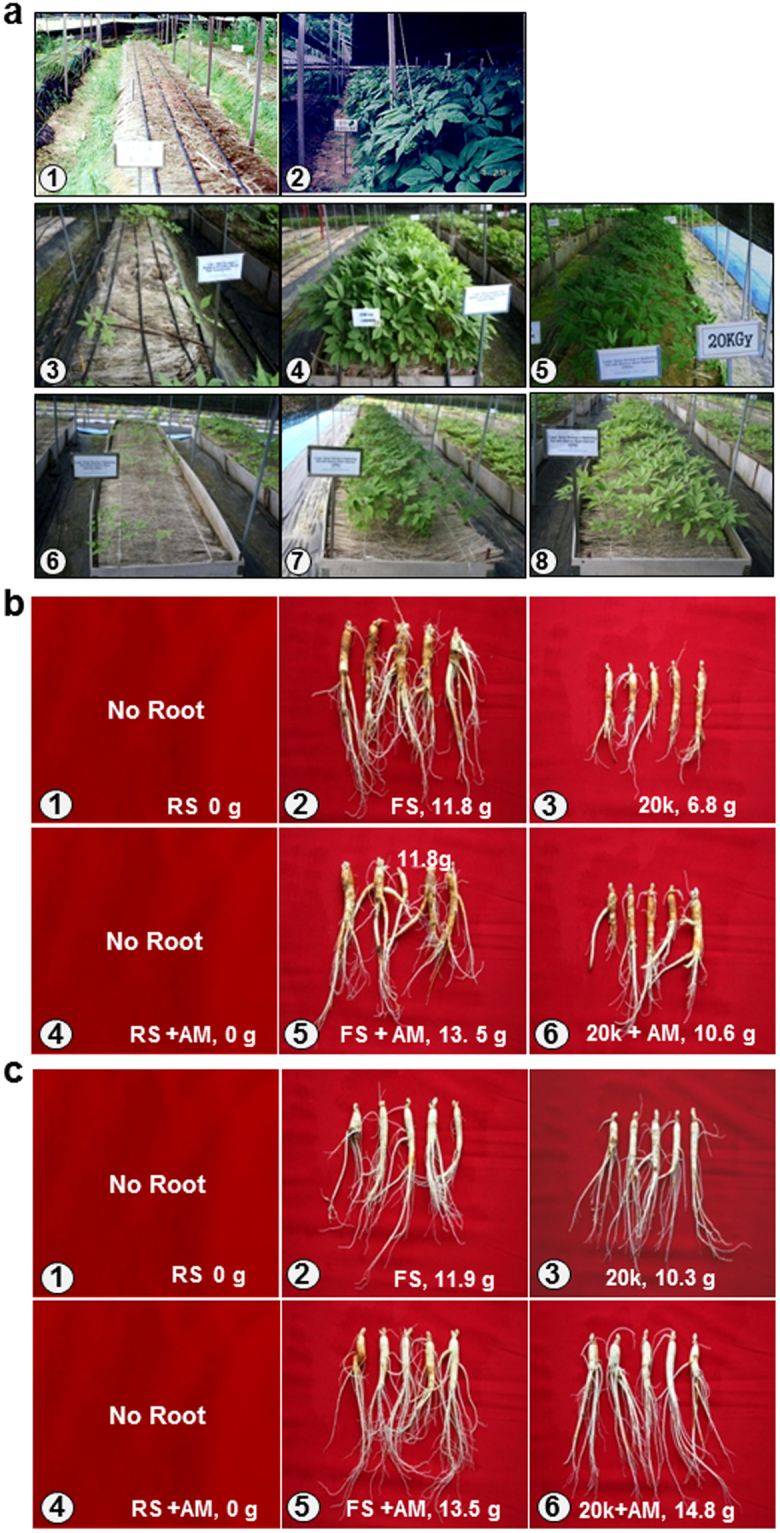
Geumsan field of 4-year-old root (4 y.) for fumigation (1997–2001). Jinan field of 3-year-old root (3 y.) and 4-year-old root (4 y.) for electron beam (EB) treatment experiments and its harvested roots (2008–2012). (**a**) ① 4 y. replanting failure (RF) in replanting soil (RS) without fumigation, ② 4 y. RS treated with fumigation, ③ 4 y. RF in RS without EB treatment, ④ 4 y. First planting soil (FS), ⑤ 4 y, RS treated with EB 20 kGy and antagonistic microorganisms (AM), ⑥ 3 y. RF in RS without EB treatment, ⑦ 3 y. FS, ⑧ 3 y. RS treated with EB treatment 20 kGy and AM. (**b**) ① No roots due to RF in RS without EB treatment, ② 4 y. roots from FS soil, ③ 4 y. roots from RS treated with EB 20 kGy, ④ No roots due to RF in RS without EB and AM treatment, ⑤ 4 y. roots from FS treated with AM, ⑥ 4 y. roots from RS treated with EB 20 kGy and AM. (**c**) ① No roots due to RF in RS without EB treatment, ② 3 y. roots from FS, ③ 3 y roots from RS treated with 20 kGy, ④ No roots due to RF in RS without EB and AM treatment, ⑤ 3 y. roots from FS treated with AM, ⑥ 3 y. roots from RS treated with EB 20 kGy and AM.

The survival rates of 6,788 roots were examined (using three replicates/treatment). The survival rate of 4-year-old root was 81.0% in RS treated with EB + AM (antagonistic-microorganisms), but 0% in untreated RS. The survival rate in EB-treated soil was comparable with that in soil used for FS (78.0%) (Fig. 1a, Table 1, and Supplementary-1 Table 1). The emergence rate of 4-year-old roots was 18.5% higher in EB-treated soil than in fumigated soil. The budding rate of 2–4-year-old roots in the second field at Jinan, which received pre-planting soil management to increase soil aggregation, was 4–6% higher than that in the first field in Jinan.

**Table 1:** Root survival rate (%).

Root Field	Soil Treatment Condition	1^st^ yr.−2^nd^ yr. old root		2^nd^ yr.−3^rd^ yr. old root		3^rd^ yr.−4^th^ yr. old root		Remark
		N/A	AM	N/A	AM	N/A	AM	
Geumsan^a^	RS + R	95.6^d^		37.0^f^		0^g^		ANOVA was carried out using the Costat. Duncnas’s multiple range test for significant differences between the treatments at p = 0.05
	RS + F	99.0^a^		84.3^d^		56.6^e^		
	RS + F + R	99.1^a^		91.0^b^		70.6^d^		
Jinan^b^ −1^st^	FS	90.3^g^	92.0^f^	81.0^e^	85.0^d^	76.0^c^	76.1^c^	
	RS	89.0^h^	89.9^gh^	5.0^l^	10.0^h^	0^g^	4.2^f^	
	RS + EB 20kGy	94.0^e^	95.7^d^	89.0^c^	84.0^d^	77.5^bc^	79.2^b^	
Jinan^c^ −2^nd^	FS	96.7^cd^	98.3^ab^	81.1^e^	85.7^d^	78.85^b^	78^bc^	
	RS	93.0^ef^	97.5^bc^	12.5^g^	35.9^f^	0^g^	0^g^	
	RS + R + EB 20kGy	97.9^abc^	93.2^ef^	88.9^c^	95.7^a^	78.9^b^	85.7^a^	

Abbreviation: RS + R: Replanting soil after R, RS + F: Replanting soil treated with fumigation, FS: First planting soil, RS + EB 20 kGy: Replanting soil treated with electron beam 20 kGy Soil, RS + R + EB 20 kGy: Replanting soil treated with electron beam 20 kGy after R, R: Planting Rye for pre-seedling soil management, AM: antagonistic microorganism, NA: No treatment with antagonistic microorganism.

^a^Oct. 1997: Harvest 6-year-old root, 1998: Soil management with fumigation, Apr. 1999: Transplant 1440 ginseng root seedlings (GS), Jun. 1999, 2000 and 2001: Survey.

^b^Nov. 2008: Harvest 6-year-old root, Feb. 2009: Soil management with EB, Mar.2009:Transplant 2492 GS, Jun. 2009, 2010, 2011 and 2012: Survey.

^c^Nov. 2008: Harvest 6-year-old root, Feb. 2009: Pre-seedling soil management with planting rye (or Sudan grass), Feb. 2010: Soil management with EB, Apr. 20:, Transplant 2856 GS, Jun. 2010, 2011 and 2012: Survey.

The growth of 4-year-old root was better in FS than in RS treated with EB+AM, and the average lengths of the roots were 26.8 cm and 18.8 cm, and the average weights were 13.5 g and 10.6 g, respectively. By contrast, in RS treated with EB+AM after pre-seedling management using rye, the 3-year-old roots were comparable with those grown in FS, with average root lengths of 22.5 cm and 26.1 cm, average tap root lengths of 5.9 cm and 6.5 cm, average tap root diameters of 14.8 cm and 16.3 cm, average fine root numbers of 14.8 ea. and 19.2 ea., average lateral root numbers of 4.0 ea. and 3.8 ea., and average fresh weights of 12.9 g and 13.5 g, respectively (Fig. 1b,c, Table 2, and Supplementary-1 Tables 2 and 3).

**Table 2:** Root growth in electron beam treated soils (cm, ea., and g).

Old	Root growth			Root Length (cm)		Tap root Length (cm)		Tap root Diameter (mm)		Number Of Fine Roots (ea.)		Number Of Lateral Root (ea.)		Fresh weight (g)	
	Microorganism			NA	AM	NA	AM	NA	AM	NA	AM	NA	AM	NA	AM
4 yr-old Root	Jinan (1^st^)	Treatment	FS	23.7^b^	26.8^a^	8.0^a^	6.8^abc^	18.9^a^	20.2^a^	19.8^a^	16.4^b^	4.4^a^	3.6^abc^	11.8^b^	13.5^a^
			RS	0.0^f^	0.0^f^	0.0^d^	0.0^d^	0.0^f^	0.0^f^	0.0^e^	0.0^e^	0.0^d^	0.0^d^	0.0^e^	0.0^e^
			RS + EB 20kGy	14.7^e^	18.0^d^	6.3^bc^	6.3^bc^	11.9^e^	14.0^d^	8.2^c^	8.8^c^	2.8^bc^	3.2^abc^	6.8^d^	10.6^bc^
3 yr-old Root	Jinan (2^nd^)		FS	23.2^b^	26.1^a^	7.5^ab^	6.5^bc^	15.4^c^	16.3^bc^	8.4^c^	19.2^a^	2.2^c^	3.8^ab^	11.9^b^	13.5^a^
			RS	0.0^f^	0.0^e^	0.0^d^	0.0^d^	0.0^f^	0.0^f^	0.0^e^	0.0^e^	0.0^d^	0.0^d^	0.0^e^	0.0^e^
			RS + R + EB 20kGy	21.6^c^	22.8^bc^	6.2^bc^	5.7^c^	13.5^d^	17.0^b^	8.4^c^	15.8^bc^	3.8^e^	4.2^ab^	10.3^c^	14.8^a^

Abbreviation: FS: First planting soil, RS: Replanting soil, RS+EB 20 kGy: Replanting soil treated with electron beam 20 kGy, RS+R + EB 20 kGy: Replanting soil treated with electron beam 20 kGy after R, AM: treatment antagonistic microorganism, NA: No treatment with antagonistic microorganism, R: Planting rye or Sudan grass for pre-seedling soil management.

Remark: ANOVA was carried out using the Costat. Duncan’s multiple range test for significant differences between the treatments at p = 0.05.

Plant diseases were less frequent in plants grown in EB-treated soil than in those grown in untreated RS and FS because the soil-borne fungal pathogens had been removed by EB (Supplementary-1 Fig. 8b and Table 4). Fungi (Alternaria solani, Botrytis cinera, Colletotrichum gloeosporioides, Fusariu*m solani*, *Phytophthora drechsleri*, *Pythium aphanidermatum*, and *Sclerotinia sclerotiorum*) inoculated into soil treated with autoclave (120 °C/20 min.) were irradiated with EB 20 kGy and their sterilization were identified by plating on potato dextrose broth agar (PDA 50 g/500 ml 25 °C). The survival of bacteria (*Bacillus amyloliquefaciens* and *Pseudomonas toraasii*) after 20 kGy EB was identified by plating on nutrient broth agar, (NBA: 50 g/500 ml 25 °C) (Supplementary-1 Fig. 8b). The rate of root rot was reduced by 10–20% when EB was combined with AM. The EB field did not contain weeds, which serve as host plants for pathogens (Supplementary-1 Fig. 5). High performance liquid chromatography (HPLC) showed that the amount and type of ginsenoside did not differ significantly between roots grown in EB-treated and those grown in virgin soils (Supplementary-1 Fig. 6 and Table 5).

### Microbial and physicochemical properties of the soil after EB irradiation

**Figure 2:**
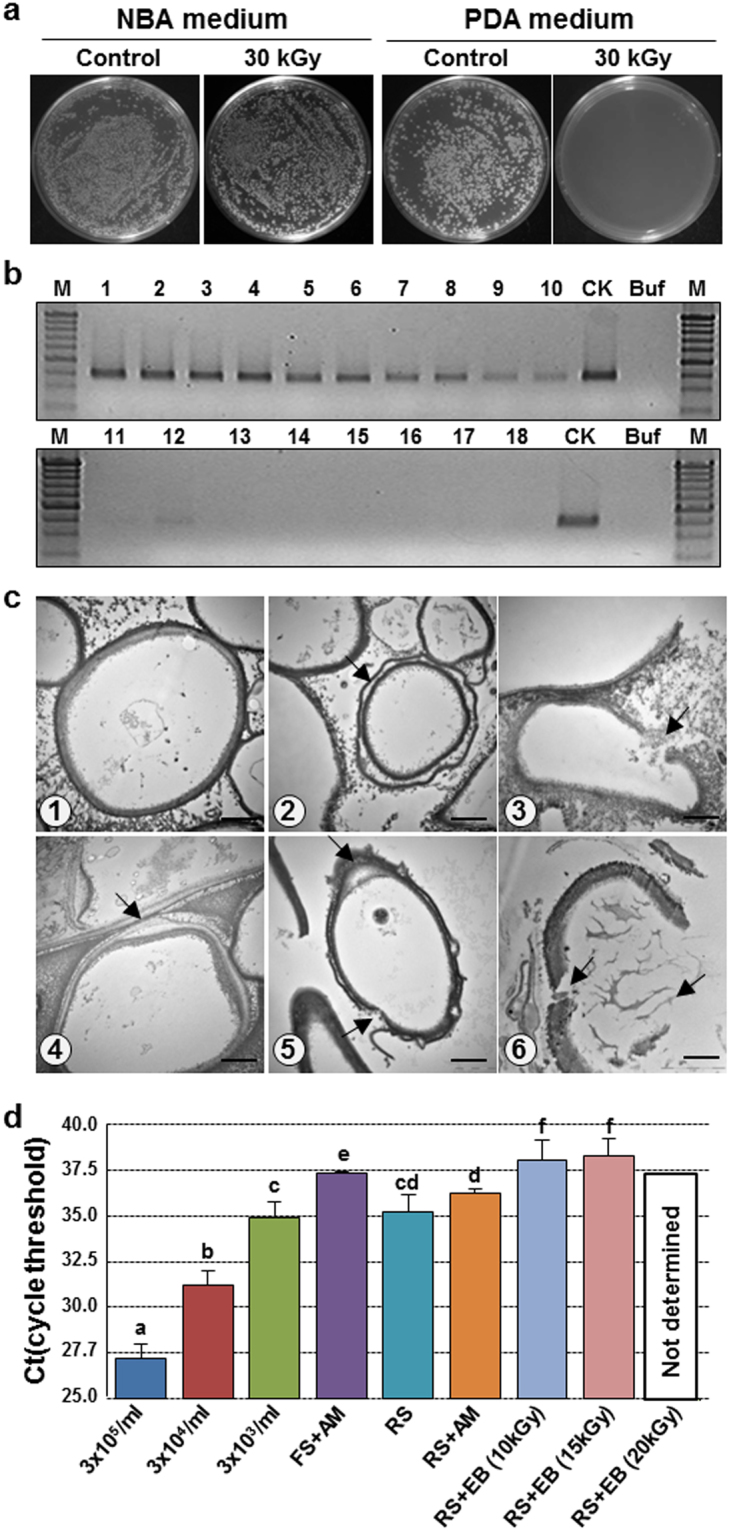
The results of EB treatment on *C*. *destructans*, fungi, *Actinomyces*, and bacteria existed in soil. (**a**) Nutrient broth agar (0.5 g/5 ml ddw 30 °C) and potato dextrose agar (0.5 g/5 ml ddw 25 °C) on fungi, *Actinomyces*, and bacteria existed in soil. Soil from field in which root was grown for 6 years. Control: Suspended soil, 30 kGy: Suspended soil treated (thickness 1 cm) with EB 30 kGy, 30 kGy in NBA medium: The viability of *Actinomyces* and bacteria, 30 kGy in PDA medium: The mortality of filamentous fungi. (**b**) Gel electrophoresis results of PCR of EB treatment on *C*. *destructans* existed in soil; Lane M: 100 bp DNA ladder, Lane CK: Control, Lane Buf: Buffer, Lane (L) 1–2: 0.5 kGy, L 3–4: 1 kGy, L 5–6: 2 kGy, L 7–8: = 4 kGy, L 9–10:10 kGy, L 11–12: 15 kGy, L 13–14: 20 kGy, L 15–16: 25 kGy, and L 17–18: 30 kGy (odd lane: thickness 0.5 cm and even lane: thickness 3 cm). (**c**) ①–⑥ TEM images of *C*. *destructans* treated with EB irradiation at 0 (control), 10, 15, 20, 30, and 40 kGy, respectively. Cell membranes and cytoplasm of chlamydospores, hypha, and conidia of *C*. *destructans* (arrowed) were ruptured. Scale bar: 2 μm. (**d**) PCR cycle threshold (Ct) value of 2011 inoculum density of *C*. *destructans* isolates formed on PCNB agar medium obtained from 2009 Jinan first root field treated with FS + AM, RS, RS + AM, and RS + EB (10 kGy, 15 kGy, and 20 kGy, respectively) based on *C*. *destructans* colonies density (3 × 10^5^/ml, 3 × 10^4^/ml and 3 × 10^3^/ml, respectively) using amplification curve analysis of SYBR Green I of real-time PCR with primer sets CDPCF12/CDPCR121 and CDIGS47NF2/CDIGS47NR1.

After exposure to ≤30 kGy EB, the mortality of filamentous fungi was determined by plating on potato dextrose broth agar (PDA), whereas the viability of *Actinomyces* and bacteria was tested on nutrient broth agar (NBA)(Fig. 2a and Supplementary-1 Fig. 8a,b). In both cases, the inoculum was the soil in which the root had been grown for 6 years.

The densities (10^5–6^ CFU/g) of filamentous fungi, *Actinomyces*, and aerobic bacteria in the 10–40 kGy EB-treated RS field were identical to those in the FS field based on a survey performed 2 months after EB treatment (Supplementary-1 Table 6). The physicochemical properties of the mineral nutrients did not change significantly after 20 kGy EB irradiation (Supplementary-1 Table 7a) and were comparable with those of the soil in which roots were grown under standard conditions (Supplementary-1 Table 7b–d).

The earthworm field test was conducted for 30 days; no differences were observed between the weights in FS soil (6.0 g) and those in EB-treated soil (6.1 g) (Supplementary-1 Fig. 7). Neutron activation analysis detected no heavy metals or aberrations after soil irradiation (Supplementary-1 Table 8).

### PCR and TEM analysis of *C*. *destructans* after EB irradiation

The sterilizing effect of EB on *C*. *destructans* was investigated by analyzing DNA extracted from eight soil types. PCR analysis of soil after irradiation showed that *C*. *destructans* was eliminated by doses of 20–30 kGy EB. However, it survived in soil after irradiation at <15 kGy (Fig. 2b). Thus, irradiation at >20 kGy eliminated *C*. *destructans* as identified by PDA/PCNB (pentachloronitrobenzene nutrient broth) (Supplementary-1 Fig. 8c,d). Transmission electron microscopy (TEM) showed (Fig. 2c) that EB or reactive oxygen species and hydroxyl free radicals of EB-generating might directly or indirectly rupture the cell membranes of *C*. *destructans* chlamydospores, hyphae, and conidia with a size greater than 10 μm, causing the release of the cytoplasm^43,44^.

The concentration of *C*. *destructans* in a field was periodically monitored by performing real-time amplification SYBR green I PCR with a specific primer set (Supplementary-1 Figs 9–11). Unknown concentrations of sampled soils were read from a standard curve obtained using 10-fold serially diluted chlamydospores (10^5^–10^3^ spores). The inoculum concentration detected in November, 2011 in the first Jinan field (March 2009) did not reach the level of 10^3^ CFU/g, that causes severe root-rot (Fig. 2d).

### NT measurement of the volume/water content of roots in soil

**Figure 3:**
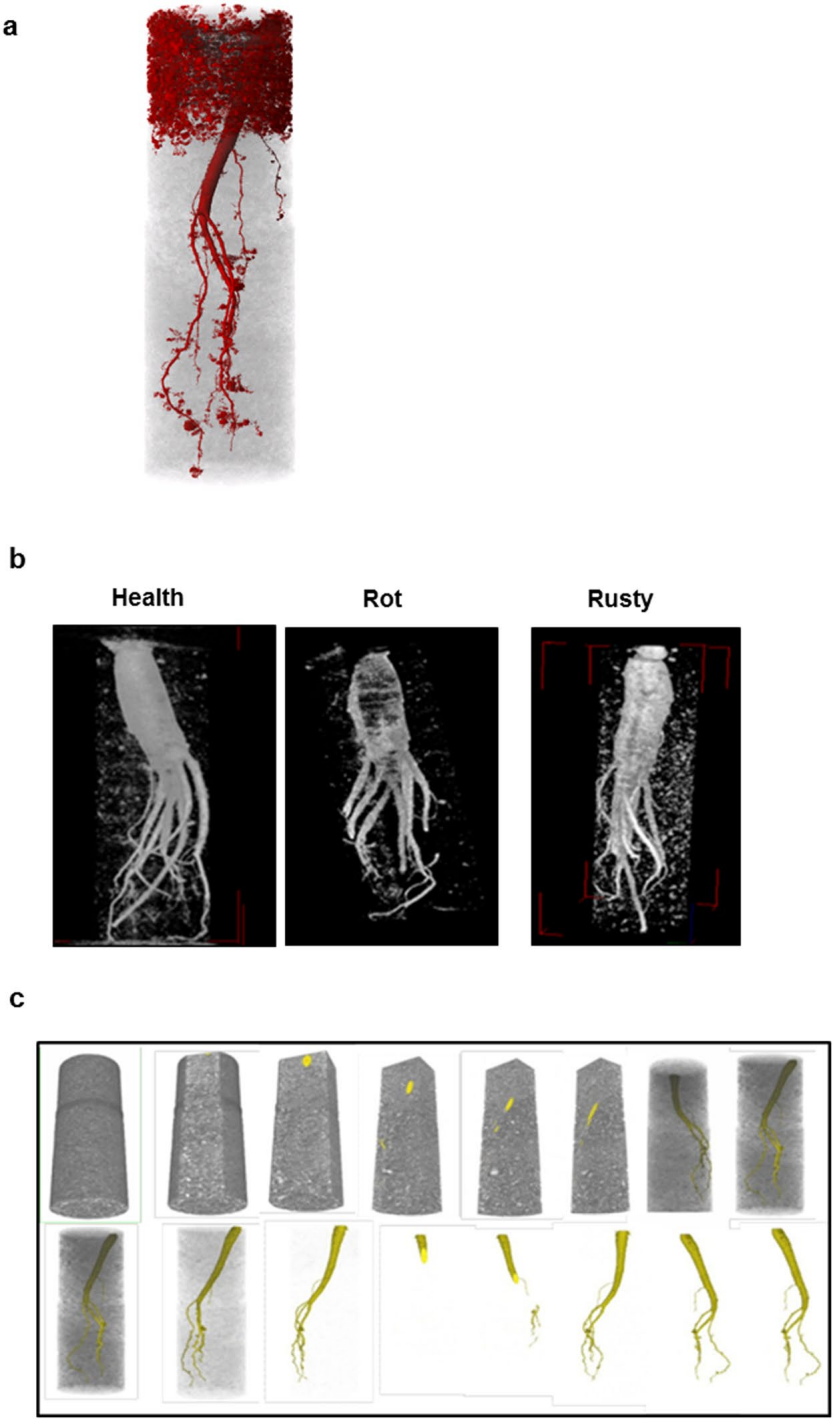
Neutron image of root planted in Al pot for diagnosing root morphology and pathology. (**a**) A root is visualized as colouring neutron image with resolution at 0.3 mm size, 4-year-old root in the 5 cm diameter soil, exposure time 10 sec/1 projection. (**b**) Healthy 4-year-old root, rotten 4-year-old root, and rusty 4-year-old root. (**c**) Images of axial slices from a neutron tomogram, which show a root embedded in the soil.

A neutron tomography procedure was developed to determine root water content and diagnose plant diseases in the soil. NT facilitated the noninvasive visualization of the root architecture *in situ* and allowed quantification of its water content during periodical checks during the winter hibernation period (**S**upplementary-2 Method 1, Supplementary-2 Figs 1 and 2). The visualization of a whole root and the 0.3 mm hairy roots in the soil is presented (Fig. 3a). Neutron imaging of healthy and rotten (caused by highly virulent *C*. *destructans*) roots was performed at a resolution of 160 µm^45^ (Fig. 3b). In contrast to the healthy plant surface^46^, the epidermal and cortical tissues of the roots were stained by rust, which was caused by weakly virulent *C*. *destructans* and *Fusarium solani* (Fig. 3b). The rust contained phenolic compounds, which yielded a different neutron attenuation coefficient from that of adjacent healthy tissues^46^. The images of axial slices from a neutron tomogram, which show the water and organic matter in a root embedded in the soil are shown (Fig. 3c).

**Figure 4:**
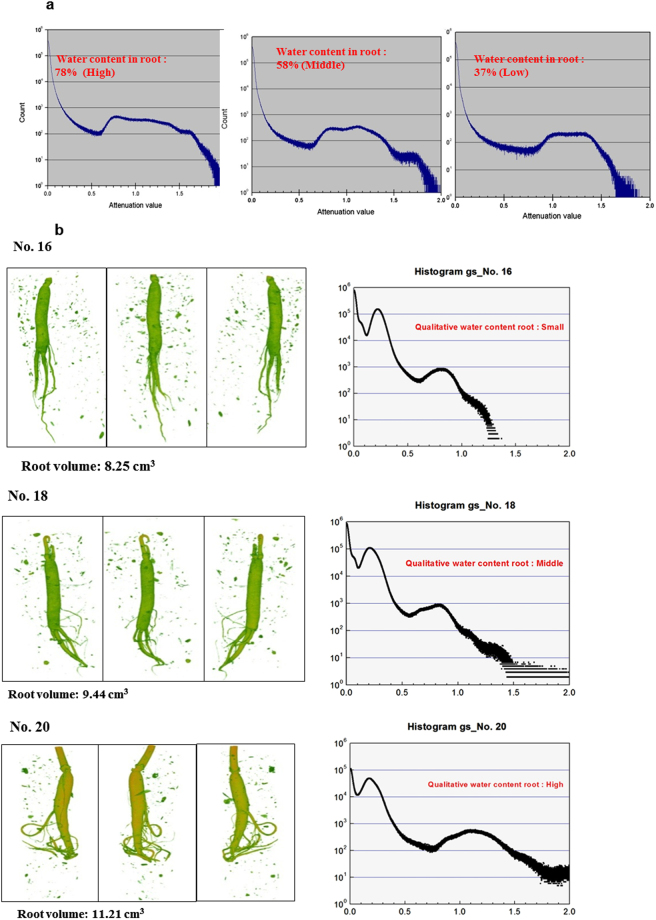
Neutron tomography image of 3 year-old roots in 6 cm diameter soil in aluminum (Al) pots embedded in Geumsan/Jinan root field for diagnosing pathology and measuring qualitative water content. (**a**) Histogram of water content in root. x axis: attenuation value, y axis: count (voxel number): Histogram of high water content (72%) in root, histogram of middle water content (58%) in root, and histogram of low water content (37%) in root. (Camera: Roper Scientific 1340 × 1300, and scintillator: 50 μm). (**b**) Neutron tomography image on Gumesan field 3-year-old root showing root volume (cm^3^), qualitative water content, and histogram (x axis: attenuation value, y axis: count = voxel number): Al pots, No. 16, No. 18, and No. 20 (Camera: Roper Scientific 1340 × 1300, and scintillator: 150 μm).

**Figure 5:**
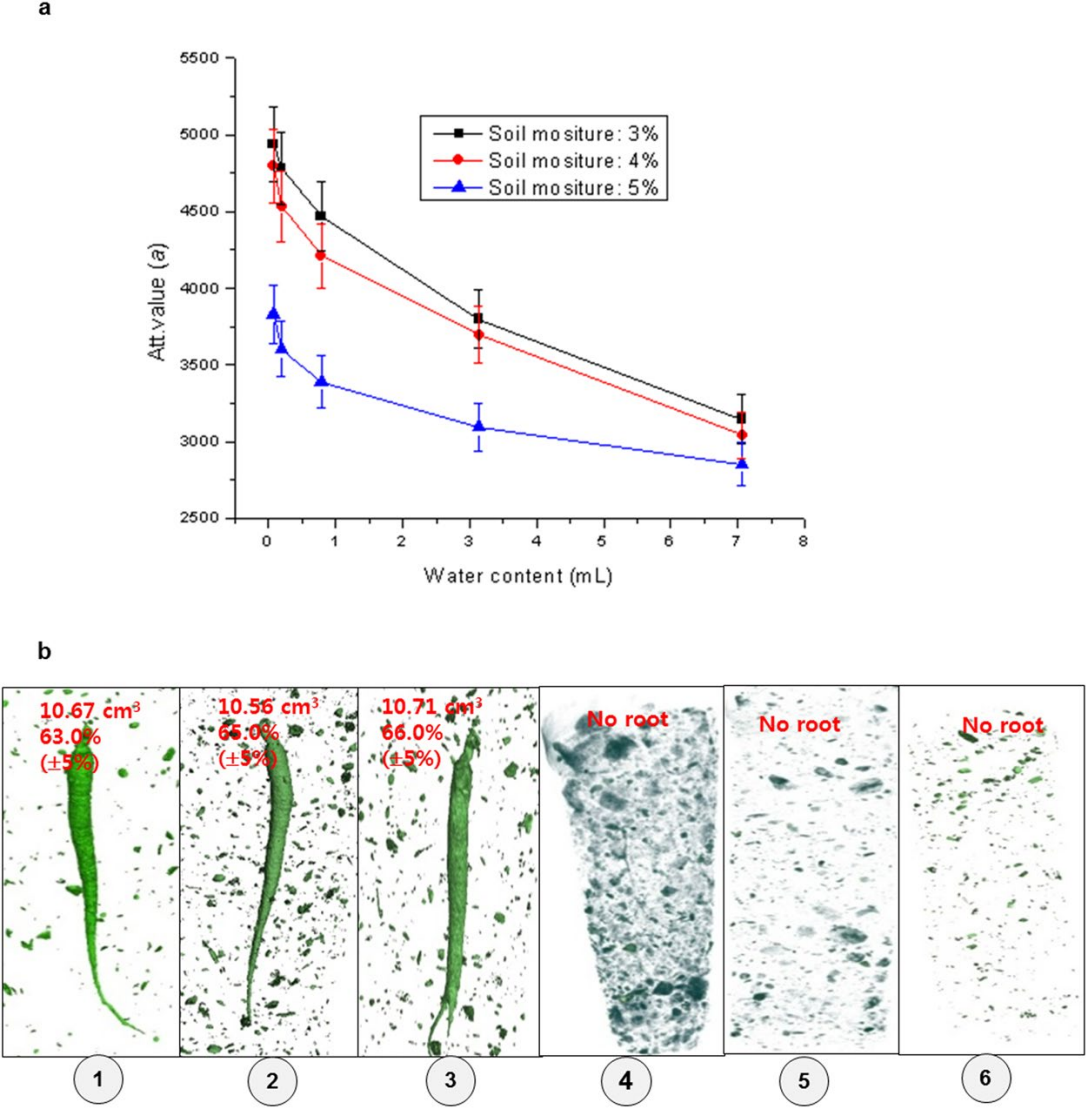
Neutron tomography image of 3 year-old roots in 6 cm diameter soil in aluminum (Al) pots embedded in Geumsan/Jinan root field for diagnosing pathology and measuring quantification water content. (**a**) The calibration curve (water amount 3%-5% in soil): water amount (x axis) vs. neutron attenuation value (y axis). (**b**) Neutron tomography ①–③ of the root volume (cm^3^)/water content (mL) of the 3-year-old roots in Al pots No.7, No.8, and No.17, which contained soil treated with EB 20 kGy, were 10.67 cm^3^/63.0% (±5%), 10.56 cm^3^/65.0% (±5%), and 10.71 cm^3^/66.0% (±5%), respectively, ④–⑥ of the root volume/water content (mL) of the 3-year-old roots in Al pots No. 100, No. 110, and No. 120, which contained untreated soil, were 0.9 cm^3^/0%, 0 cm^3^/0%, and 0 cm^3^/0%, respectively.

The roots were 3D-imaged and their weights were physically measured outside the soil in both their saturated and dry states. This comparison was found to be off from the weight measurements by 5% due to neutron scattering effect. The histogram of 72% (high), 58% (middle), and 37% (low) water in root was illustrated for measuring relative water content qualitatively (Fig. 4a). Aluminum phantoms of varying diameters filled with water were embedded in moist soil conditions of 3–5% to generate a calibration curve for quantifying the water level (Fig. 5a).

In 2008, seven 3-year-old roots in soil from the Geumsan field were 3D-imaged with NT to measure the water content qualitatively. The roots in Al pot 20 had a higher water content than those in Al pots 16, and 18 with root volumes of 8.25–11.46 cm^3^ (Fig. 4b). In 2010, 3-year-old roots from the Jinan field were imaged with NT. The quantification of root volume/water content of the 3-year-old roots in Al pots 7, 8, and 17 treated with EB 20 kGy, were 10.67 cm^3^/63.0% (±5%), 10.56 cm^3^/65.0% (±5%), and 10.71 cm^3^/66.0% (±5%), respectively (Fig. 5b ①–③). By contrast, the volume/water content in Al pots 100, 110, and 120, containing untreated soil, were 0.9 cm^3^/0%, 0 cm^3^/0%, and 0 cm^3^/0% respectively; this was due to root-rot disease (Fig. 5b ④–⑥).

## Discussion

In the study, EB cultivation techniques and NT are proved to conserve and continually produce endangered rare species, such as *Astragalus*, *Dioscorea*, *Glycyrrhiza*, *Harpagophytums*, *Panax ginseng*, *Rauvolfia*, and *Valeriana*. The use of herbal medicines is anticipated to increase globally. This is due to the increasing size of the global population and to consumer preference for natural and eco-friendly medicines over synthetic drugs. These methods allow continual cropping on the same soil without pesticide treatment. This is a major step toward the environmentally friendly production of endangered therapeutic herbs.

### Electron beam treatment on soil

The study showed that treatment of pre-used soil with EB and AM yielded a survival rate of 81% in 4-year-old root. By contrast, the survival rate in untreated used soil was 0%. ; the treatment virtually eliminated *C*. *destructans* and other soil-borne pathogens whereas *Actinomyces* and aerobic bacteria became prevalent shortly after EB treatment. *Actinomyces* and bacteria survived in soil after irradiation because they were less sensitive than fungi to the free radicals released by water radiolysis, due to their size^27^. In November, 2011, *C*. *destructans* inoculum concentration in the first Jinan field constructed on March, 2009 did not reach the level of 10^3^ CFU/g; therefore, the level of root rot was not as severe as in 2011^14^. The occurrence of various plant diseases results in crop yields of <60% for 6-year-old root in FS fields^47^. Thus, the use of real-time PCR to diagnose the diseases of foliage and soil-borne is essential. The pH of the treated soil (pH 5.6) tended to be slightly higher than the soil pH (5.5) recommended by the physicochemical properties guidelines for root plant cultivation **(**Supplementary-1 Table 5a–c); therefore, sulfur may be required to reduce the pH. The root growth in EB soil was comparable with that in the FS field. Pre-seeding soil management was required to increase soil aggregation and ensure optimum growth. Root growth in EB soil was comparable to that in the FS field. The success of the Jinan field experiments, which showed that secondary planting in the same soil could be achieved without chemical treatment, is a major advance. The need to move continuously among fields to avoid replanting failure limits the amount of available cultivation land because that ginseng plant is mainly suited to silty loam soil weathered from granite, gneiss, and basalt at northern/southern latitudes of 34–45°. Treatment with EB facilitates ongoing cultivation without the need for virgin land, thus allowing standardization of settlement cultivation, and increases productivity on the endangered phytotherapeutic rarity-roots (Supplementary-1 Fig. 12a, b). Instead of using muslin to produce artificial shade, photovoltaic panels with 10–30% transparency can be used in the ginseng settlement fields for enhancing sustainability of renewable energy to maintain their facility (Supplementary-1 Fig. 12a,b).

The experiment was conducted with 2.5 MeV × 100 kW EB to accelerate electrons in a linear direction^48^. A microtron is available now that accelerates electrons in a circular direction^49^. This configuration maximizes both the use of space and efficiency because it has a diameter of 1.0 m and a height of 0.8 m, with an output of 4 MeV × 40–100 kW EB. This development will facilitate its application in the cultivation field *in situ* with an unprecedented beam enhancement provided nanotechnology^50^. In practice, the mass of soil throughput for an EB facility of 2.5 MeV × 100 kW is 11.1 t/h at 20 kGy (Supplementary-1 Note 1), which optimizes the balance between reducing soil-borne pathogens and retaining soil-microbial characteristics and enzyme activity. A single unit EB facility can treat an area of 118,000 m^2^/year in case of a cultivation soil depth of 30 cm *in situ* (Supplementary-1 Note 1). Thus, a field of ≥1,000,000 m^2^ could be processed, which is suitable for pesticide-free perennial root plant cultivation in a growing period of at least, 4–6 year. Sterilizing of soil with 20 kGy irradiation does not produce adversely affect conditions of vesicular-arbuscular mycorrhiza^51^. In the case of a ginseng root field with a cultivation soil depth of 30 cm, economically efficient organic cultivation based on EB soil treatment is feasible for areas exceeding 15,000 m^2^ *in situ* (Supplementary-1 Note 2 and Supplementary-1 Note 2 Figs 1–3). Therefore, EB treatment *in situ* is beneficial for the cultivation of medicinal herbs with a shallow depth of cultivation soil (≤30 cm).

The soil of nursery medium used in protected cultivation becomes severely contaminated by pathogenic fungi after only two or three uses. Flocs of antibiotic resistant strains flourish in these freshwater systems^52^. Thus, the recycling of nursery medium and freshwater after EB sterilization could facilitate urbanized cultivation system and the hygienization of aquatic environments^28,53^. EB Treatment on food of improving its microbiological safety and storability is one of challenging applications^54–56^. Soil is easily sterilized with EB; therefore, this technique is a viable alternative to culture in hydroponics. Thus, electron beam soil management could facilitate the cultivation of pesticide-free crops and continuous cropping in the same fields. Especially, soil electron beam treatment will allow the use of soil medium in huge protected cultivation area without any of difficulties associated with aquaculture. EB sterilization is likely to be suitable for the production of many of the valuable herbs that are susceptible to diseases associated with continuous cropping. The therapeutic herbs are threatened with extinction owing to their excess-exploitation and to soil contamination by root rot pathogens. Sterilization of soil with EB will help to conserve these species and enable them to be produced in sufficient quantities to satisfy increasing demand. We expect that this technique developed in this study will be suitable for the production of other valuable herbs are also susceptible to continuous cropping diseases including *Angelica sinensis*, *Astragalus membranaceus*, *Cnidium officinale*, *and Mandragora officinarum*.

### Neutron tomography

NT measurements of roots in the Jinan field showed a water content of >60%, which indicated healthy conditions^47^. Therefore, neutron tomography is the most appropriate method for studying the epidemiology of root-rot and rust because it can detect significant accumulations of inorganic elements of iron, aluminum, silicon, and magnesium ions and water of root in the soil, all of which interact with the fungi, mycorrhiza, and yeast inocula in the rhizosphere^13,14,45,46,57^. The levels of water, phenolics, and inorganic elements in the roots are all indicators of root health^47,57^.

The uptake of water and inorganic elements by roots is a crucial process for plant health. Dielectric cell pressure probes, magnetic resonance, and heat tracing can be used to map the fluid dynamics in the xylem and phloem, but they are destructive methods^58^. By contrast, neutron dynamic tomography^59^ produces a 3D picture of water movement in the vessels and sieve tubes, depending on solution ion concentration, pH, root pressure, osmotic pressure, capillarity, and nonpolar solvents during active metabolism and photosynthesis^60^. Water movement from the root epidermis to the endodermis, apoplast, symplast, and transmembrane regions can be analyzed. Neutron imaging with the contrast agent, D_2_O, can be used to visualize *in situ* photomorphogenesis in the plant roots based on the sensitivity to different light wavelengths^61,62^. These phenomena are largely uncharacterized at present^63^. Small neutron source other than fission reactor type is a diverse array of application in area of prostate cancer treatment^64^, petroleum exploration, detection of explosive material^65^, and inspection of aeronautics and astronautics components^66^. This study utilized a neutron source of fission reactor. The result will promote the utilization of neutron imaging with compact neutron generator for application in plant biology and agriculture production.

Conclusively, the combination of EB, and NT along with biological-controls and molecular-identification could facilitate improvements in root-crop cultivation and ensure the availability of phytotherapeutics in the 21^st^ century. This approach based on physically engineering provides novel solutions for the conservation of endangered medicinal plant while at the same reducing the environmental load of pesticide contamination. It will also increase the affordability of high-quality phytotherapeutic herbs by providing an ecologically sustainable solution to continual cropping in the same soil.

## Methods

### Electron-beam

Type = ELV-8, energy = 2.5 MeV; beam power = 100 kW; beam current = 12 mA; soil exposure throughput = 10 t/h. In this type of machine, electrons are injected from an external source into an evacuated waveguide that is excited by a high intensity electromagnetic field using a klystron that operates at microwave frequencies in the cavity resonators. Energy gain of 2.5 MeV is used for sterilization of soil borne pathogens. The physical interaction of electron beam leads to loss of energy of radiation and production of ionization and excitation of molecules of pathogens which convert into free radicals in pico to femto seconds after physical interaction with atoms. Chain reactions also, particularly play a role in damage to cell membranes of soil pathogens. The hydroxyl free radicals rupture the cell membranes of *C*. *destructans* chlamydospores, hyphae, and conidia with a size greater than 10 μm, causing the release of the cytoplasm.

### Dosimetry System

ISO/ASTM 51650: Practice for use of a cellulose triacetate (CTA) dosimetry system.

### Medicinal-root fields used for fumigation and EB-treatments

The characteristics of Geumsan field size = 100 m^2^; number of transplanted root seedlings used in each of three replicate tests = 1440; period = 1997**–**2001. The characteristics of 1^st^ Jinan field size = 200 m^2^; number of transplanted root seedlings used in each of three replicate tests = 2492; period = 2008–2012. The characteristics of 2^nd^ Jinan field size = 300 m^2^; number of transplanted root seedlings used in each of three replicate tests = 2856; period = 2008–2013. (Fig. 1 and Supplementary-1 Figs 1 and 2).

### The root field used for neutron tomography trials

(Supplementary-2 Fig. 1 and Method 1). Geumsan field: 2–3-year-old root plants were transplanted to cylinder-type aluminum pots (50) embedded in the soil during 2002–2006. 1^st^ Jinan field: root seeds were sowed in aluminum pots (600) embedded in the soil during November 2007–November 2008. 2^nd^ Jinan field: root seedlings were transplanted to aluminum pots (400) embedded in the soil during 2008–2012.

### Root species

*Panax ginseng* C. A. Meyer.

For many thousand years, mankind has been using *Panax ginseng* C. A. Meyer as nutrient, beverage, cosmetics, dye and medicine to maintain health and to improve quality of life. In Asia, particularly, *Panax ginseng* C.A. Meyer is considered to be the most precious plant among herbs, and ginseng has been in the spotlight worldwide. Even in the Western world, where there are greatly advanced research facilities and highly qualified man-power available, and are regarded to be capable of conquering any hard-to-cure ailments, many peoples has recently been reported to use herbal medicine, particularly ginseng. The genus name *Panax* (Pan = all + axos = medicine) means ‘cure all’ in Greek. (Yun,T.K. Brief Introduction of *Panax ginseng* C.A. Meyer *J Korean Med Sci*; **16(Sup.):** S3–5 (2001)).

### Ginseng root variety/breed

*Cheon Poong* variety of *Panax ginseng* C. A. Meyer.

### Fungi/Bacteria

*Cylindrocarpon destructans*, *Alternaria solani*, *Botrytis cinerea*, *Colletotrichum gloeosporioides*, *Fusarium solani*, *Phytophthora drechsleri*, *Pythium aphanidermatum*, *Sclerotinia sclerotiorum*, *Bacillus amyloliquefaciens( + )*, *and Pseudomonas tolaasii(-)* were obtained from CNU and KAMRC.

### Antagonistic microorganism

*Bacillus subtilis 1:1500*.

### Phylogenetic analysis of *C*. *destructans*

(Supplementary-1 Fig. 11). The phylogenetic tree was reconstructed using the neighbor-joining algorithm based on the distances calculated using Kimura’s two-parameter model with the sequences DQ831947, DQ831946, AY380917, AF315209, AF315207, AF315205, AF315197, AF315200, AF315205, AF315198, AY997578, AY997580, AY997576, AY997579, AY997577, AY997581, AF315203, AF315204, and AF3152035 from GenBank.

### DNA extraction

Distilled water (400 μL) was added to 0.2 g soil, mixed with glass beads, and ground with 300 μL extraction buffer (200 mM NaCl, 200 mM Tris-HCl [pH 8], 30 mM EDTA, and 0.5% SDS) and 300 μL CTAB buffer (1.4 M NaCl, 100 mM Tris-HCl [pH 8], 20 mM EDTA, and 1% polyvinylpyrrolidone). The mixture was extracted with chloroform-isoamyl alcohol (24:1) by vortexing and centrifuged. The supernatant was precipitated with two volumes of 100% ethanol, centrifuged, and purified using a minispin column (G-spin kit, Intron).

### PCR

Internal transcribed spacers (ITSs) were amplified using the oligonucleotides ITS1 (5′-TCCGTAGGTGAACCTGCGG-3′) and ITS4 (5′-TCCTCCGCTTATTGATATGC-3′). The first PCR reactions were performed in a volume of 50 μL containing 5 ng DNA, 0.4 μM each of the ITS1 and ITS4 primers, 250 μM deoxynucleoside triphosphates, 50 mM KCl, 1.5 mM MgCl_2,_ and 1 unit of Taq DNA polymerase (Takara Biomedicals Co., Japan). The cycle comprised an initial denaturation at 95 °C for 3 min, followed by 35 cycles of 95 °C for 35 s, 55 °C for 1 min, and 72 °C for 2 min. The reaction was completed by a final extension at 72 °C for 8 min. Dest1 (5′-TTGTTGCCTCGGCGGTGCCTG-3′) and Dest4 (5′-GGTTTAACGGCGTGGCCGCGCTGTT-3′) primers were used in the two-step nested PCR and were specific to *C*. *destructans*. One microliter of the PCR product from the first amplification was used. The PCR products were separated on a 1% agarose gel. The real-time amplification curve of SYBR green I PCR (C1000, Bio-Rad) was constructed to quantify *C*. *destructans* DNA using the specific primers CDPCF12 and CDPCR121. The reaction conditions were the same as those described above, except 40 cycles were used and the annealing temperature was 60 °C.

### Soil analysis

The pH meter (EUTECH COND600) and EC meter (EUTECH ECOSCAN) analyses used one part dried soil to five parts water (w/v). The Tyurin method was used to determine the organic matter content: 0.2 g soil was passed through a 0.25 mm sieve and was heated together with 10 mL 0.4 N K_2_Cr_2_O_2_ at 200 °C for 5 min, before adding 50 mL water and 5 mL H_3_PO_4_ containing diphenylamine, followed by titration using 0.2 N Fe(NH_4_)_2_(SO_4_)_2_. The Kjeldahl method (Kjeltec 2400-Analyzer, Foss) was used to determine the nitrogen content: 1 g soil and 15 mL H_2_SO_4_ were heated with the catalyst CuSO_4_ at 420 °C for 50 min. The Lancaster method (HP8453 UV-VIS, Agilent) was used to determine available phosphate: 5 g soil and 20 mL CH_3_COOH were shaken and filtered through a No. 2 filter paper. The chromophore level was analyzed by heating 3 mL of the filtrate with 6 mL ammonium paramolybdate and 0.4 mL 1-amino-2-naphtol-4-sulfonic acid at 30 °C for 30 min, before reading the absorbance at 720 nm. Inductively coupled plasma-mass spectrometry (GBC, Intergra) was used to determine the exchangeable cations Ca^2+^, Mg^2+^, K^2+^, and Na^2+^ after shaking and filtering (No. 2 filter paper) 5 g soil and 50 mL 1N-CH_3_COOH/NH_4_OH (pH 7.0).

### Potato dextrose broth agar

Diluted soil samples (200 μL) were spread on various potato dextrose agar plates. D: original extract (0.5 g/5 mL water); 10^−1^: diluted 10-fold (1 ml D solution/9 mL double-distilled water); 10^−2^: diluted 100-fold; and 10^−3^: diluted 1000-fold. Plates were incubated at room temperature for 3 days.

### Pentachloronitrobenzene nutrient brothagar

PCNB agar was prepared from 5 g Bacto, 5 g peptone, 20 g agar, 250 mg MgSO_4_, 500 mg KH_2_PO_4_, 200 mg PCNB, 50 mg penicillin G, 1.3 mL 85% lactic acid, 20 mL ethanol, 130 mg Na desoxycholate, and 1 L water.

### Nutrient broth agar

Soil dilutions (200 μL) were spread on different plates. D: Original extract: 0.5 g/5 mL water. Plates were incubated at room temperature for 3 days.

### HPLC

Samples were analyzed by HPLC (Agilent Technologies 1200 series, USA; UV detector, 203 nm) using a C-18 reverse phase column (Zorbax Eclipsed Plus, 3.5 μm, 4.6 mm × 150 mm) and a solvent gradient system, as described in Table 3. The mobile phase comprised water (solvent A) and acetonitrile (solvent B), and the flow rate was 1.6 mL/min. Ginsenosides were identified based on comparisons of their retention times with those of authentic samples, which were confirmed using a UPLC/TQD (Ultra Performance Liquid Chromatography/Triple Quad Mass Spectrometer) (Waters, USA). Root was cultivated in Jinan, Korea and was harvested in September 2011. Most of the solvents and reagents were purchased from Sigma-Aldrich (Seoul, Korea). The HPLC eluents, acetonitrile, and deionized water were purchased from J.T. Baker. The standard ginsenoside materials were purchased from KFDA (Korea), ChromaDex (USA), and Tauto (China).

**Table 3:** Conditions used for HPLC analysis.

Time(min)	Solvent A (%) a)	Solvent B (%) b)
0	82	18
10	80	20
32	68	32
40	55	45
55	45	55
56	10	90
60	10	90

a) Solvent A: water; b) solvent B: acetonitrile.

### Neutron activation analysis

Soil samples irradiated by NAA #1 irradiation hole with a Pneumatic Transfer System (PTS) of the HANARO research reactor. The thermal neutron fluxes measured at the NAA #1 hole were up to 3.85 × 10^13^/cm^2.^sec. For the detection of short-lived and long-lived nuclides, the samples were irradiated for 3 s and 30 min, respectively. Al-01%Au and Fe monitors were co-irradiated with the samples to monitor the thermal neutron flux during the sample’s irradiation. Gamma-rays from the irradiated samples were acquired using a HPGe detector (EG & G ORTEC, 25% relative efficiency, FWHM 1.85 keV at 1332 keV of ^60^Co) coupled to a 16K-Multichannel Analyzer. Finally, the elemental concentrations in the samples were calculated by a software program developed for an absolute quantification of neutron activation analysis.

### Earthworm field test

According to the OECD test guidelines (1984), the field test used a container that measured 0.55 m (length) × 0.35 m (width) × 0.15 m (height), and 17 kg soil with a moisture content of 15%. The treatments comprised control, EB 10 kGy, EB 15 kGy, and EB 20 kGy, each with 20 earthworms for 30 days.

### TEM

For analysis, fungal samples were fixed for 2 h at 4 °C in 50 mM sodium cacodylate buffer (pH 7.4) containing 4% paraformaldehyde (v/v) and 0.1% glutaraldehyde (v/v). After washing with buffer, the samples were dehydrated using a graded ethanol series (50%, 70%, 90%, 95%, and 100%, each for 10 min), and embedded in London Resin White (London Resin Co., London, UK). Ultrathin sections (70 nm-thick) were collected on uncoated nickel grids (300-mesh) and stained with 4% uranyl acetate (w/v) and 0.4% lead citrate (w/v) before TEM (Jeol JEM-1400) at 80 kV.

### Neutron tomography

(Supplementary-2 Fig. 2). The measurements were conducted at the thermal neutron imaging facility of the HANARO reactor (Supplementary-2 Fig. 2). This facility has a mean energy of 14 meV ± 0.2 meV and intensity of 5 × 10^7^n.cm^−2^.s^−1^. The detection system is based on a CCD camera of VersArray-1300FB with an 85 mm lens and uses a Li^6^ based scintillator with a thickness of 150 μm (by 2009) and 50 μm (2010-Present) of PSI to convert neutrons to visible light with a wavelength of 440 nm. The exposure time was 20 sec./projection. The scan resulted in a total of 200 sequential images at rotation of 180° with scanning parameters of 0.2279 mm for the slice thickness. The field of view is 250 mm × 250 mm and the resulting images are 1340 × 1300 pixels. These parameters yield a pixel spacing (i.e., x-spacing and y-spacing) of 0.16 mm, a limitation related to spatial resolution. The tomographic volume was reconstructed using filtered back-projection algorithm using software tool Octo pus version 8.0 version and visualized by 3-D volume rendering software, VG Studio Max version 2.0.

### Statistical analysis

The study used a split plot design. The growth measurements were expressed as the mean ± standard deviation. ANOVA was conducted using CoStat. Duncan’s multiple range test was used to test for significant differences between treatments at *p* < 0.05.

## Electronic supplementary material

Supplementary Information

Movie 1

Movie 2

Movie 3

Movie 4

Movie 5

Movie 6

Movie 7
