# Supplementary material for: Continuous cropping of endangered therapeutic plants via electron beam soil-treatment and neutron tomography
Source: Sci Rep. 2018 Feb 1;8:2136. doi: 10.1038/s41598-018-20124-7 (PMC5794772; doi:10.1038/s41598-018-20124-7)
Supplement: Supplementary file 1 — Supplementary Information [file 41598_2018_20124_MOESM1_ESM.pdf]

Supplementary-1 Information (Electron Beam)

Continuous cropping of endangered therapeutic plants *via* electron beam soil-treatment and neutron tomography

Cheul Muu Sim[1, 4], Bong Jae Seong[2], Dong Won Kim[3], Yong Bum Kim[4], Seung Gon Wi[5], Gyuil Kim[6], Hwasuk Oh[1,7], TaeJoo Kim[1], Byung Yeoup Chung[1], Jeong Young Song[8], Hong Gi Kim[8], Sang-Keun Oh[8,9], Young Dol Shin[10,11], Jea Hwan Seok[8,,10], Min Young Kang[10], Yunhee Lee[10,12], Mabuti Jacob Radebe[13], Nikolay Kardjilov[14]& Bernd Honermeier[15]

1. *Korea Atomic Energy Research Institute, 1045 Daedeokdaero Yuseong-gu, Daejeon, 303-353, Korea.*
2. *Geumsan Ginseng & Medicinal Crop Experiment Station, Chungnam, 312-831, Korea*
3. *Specialized Crop Research Institute, Jinan gun, Jeonbuk, 567-807, Korea*
4. *National Institute of Horticulture & Herb Science, Bisani 80, Eumseong, Chungbuk, 389-873, Korea*
5. *Bioenergy Research Institute, Chonnam National University, 300 Yongbong-dong, Buk-gu, Gwangju, 500-757, Korea*
6. *Institute of Jinan Red Ginseng, Jinan gun, Jeonbuk, 567-801, Korea*
7. *RIC for Next Generation Industrial Radiation Technology, Wonkwang University. 460, Iksan-daero, Iksan-si, Jeollabuk-do, 54538, Korea*
8. *Chungnam National University, 220 Gung-dong, Yuseong-gu, Daejeon, 305-764, Korea*
9. *Plant Genomics and Breeding Institutes, Seoul National University, Gwanak-gu, Seoul 151-921, Korea*
10. *GBioMix Institute, 723-1, 2 Palbok-dong, Deokjin-gu, Jeonju, 561-844, Korea*
11. *Chonbuk National University, 567 Baekje-daero, Deokjin-gu, Jeonju, 561-756, Korea*
12. *King's College London, Palace Road, London, SE1 7EH, U.K*
13. *Nuclear Energy Corporation South Africa, 0001 Pretoria, South Africa*
14. *Helmholtz Zentrum Berlin, 14109 Berlin, Germany*
15. *Justus Liebig University Gießen, Schubertstr. 81, D-35392 Gießen, Germany*

Correspondence and requests for materials to C.M.S (cmsim@kaeri.re.kr/cmsimkaeri@msn.com) or B.H (Bernd.Honermeier@agrar.uni-giessen.de)

Supplementary-1 Information Legend

Supplementary-1 Figures

Supplementary-1 Figure 1. 3-4-year-old root planted in pots (2007-2008) for radiation soil treatment experiment. **(a)** One month of growth after gamma irradiation on soil, 5 kGy, May 2007. ① Irradiated first planting soil (FS), ② Irradiated replanting soil (RS), ③ Non-irradiated RS, ④ Non-irradiated FS. **(b)** One month of growth after EB irradiation on soil, 20 kGy, June 2008. ① Irradiated RS, ② Non-irradiated RS.

Supplementary-1 Figure 2. Root field (1997-2012) for experiment of treatment of fumigation, electron beam (EB), and antagonistic microorganism (AM). **(a)** Geumsan 4-year-old root (4y.) field (1997-2001), RS (4y.): Replanting soil and RS+F (4y.): RS treated with fumigation (June 2001). **(b)** Jinan 1st 4y. field (2008-2012: Harvested 6-year-old root on 2008, seedling transplant on Apr. 2009, 4y. on June 2011); RS (4y.): Replanting soil, FS (4y.): First planting soil, EB 10k+AM(4y.): Replanting soil treated with EB 10 kGy and AM, EB 15k+AM (4y.): Replanting soil treated with EB 15 kGy and AM, and EB 20k+AM (4y.): Replanting soil treated with EB 20 kGy and AM. **(c)** Jinan 2nd 3-year-old root(3y.) field (2008- 2012: Harvested 6- year-old rootroot on 2008, pre-seedling management with rye on 2009, seedling transplant on Apr. 2010, 3y. on June 2011); RS (3y.): Replanting soil, FS(3y.): First planting soil, EB 10k+AM (3y.): RS treated with EB 10 kGy and AM, EB 15k+AM (3y.): RS treated with EB 15 kGy and AM, EB 20k+AM (3y.): RS treated with EB 20 kGy and AM, EB 25k+AM (3y.): RS treated with EB 25 kGy and AM, EB 30k+AM (3y.): RS treated with EB 30 kGy and AM, and EB 40k+AM (3y.): RS treated with EB 40 kGy and AM.

Supplementary-1 Figure 3. 3-4 year-old root roots growth in replanting soil (RS), first planting soil (FS), first planting soil treated with AM (FS+AM), 0-40 k= RS

treated with electron beam (EB) 10 kGy-40 kGy, and 10-40 k+AM: RS treated with electron beam (EB) 10 kGy-40 kGy and antagonistic microorganism(AM). **(a)** Jinan First root field (2009 – 2012:RS soil treated EB on Feb. 2009, transplant root seedlings on Apr. 2009, and harvest 4 year-old roots on Nov. 2011). **(b-c)** Jinan second root field (2008 – 2011: Plant rye for soil management on 2009, RS soil treated with EB on Feb. 2010, transplant root seedlings on Apr. 2010, and harvest 3 year-old root on Nov. 2011).

Supplementary-1 Figure 4. The soil (100 tons) from fields in which roots were already grown for 6 years and harvested was carried to the EB facility to be treated by EB. **(a)** Jinan field after roots were grown for 6 years and harvested. **(b)** The pre-used soil carried to the EB facility. **(c)** The pre-used soil conveyed to the EB facility exposure nozzle. **(d)** Root field wood frame (1.0 m (W) x 5.0 m (L) x 0.5m (H)) for electron beam experiment.

Supplementary-1 Figure 5. The weeds of *Stellaria aquatic, Chenopodium album, Alppecurus aequalis* and *Rorippa island* were not grown in irradiated soil. **(a)** Red circle: The weeds were grown in first planting soil (FS) field. **(b)** The weeds were not grown in replanting soil (RS) field treated with EB 15 kGy.

Supplementary-1 Figure 6. HPLC analysis of root ginsenosides extracts. **(a)**The major ginsenosides standards of roots. **(b)** The ginsenosides of roots from virgin soil and replanting soil. **(c)** The ginsenosides of roots from treated RS with EB (10 kGy, 20kGy, and 30kGy, respectively). Ginsenoside standards 1:Rg1, 2:Re, 3:Rf, 4:Rg2+Rh1, 5:Rb1, 6:Rc, 7:Ra1, 8:Rb2, 9:Rb3, 10:Rd, and 11:Rg3.

Supplementary-1 Figure 7. Earthworm field test on EB treatment experiment and its control [1-2].

Supplementary-1 Figure 8. Sterilization of *Cylindrocarpon destrutants*, Fungi, *Actinomyces,* and bacteria with 10 kGy-30 kGy EB were determined by plating on PDA, NBA, and PCNB-agar. **(a)** The viability of *Actinomyces* and bacteria existed in used soil (for 6 years) with EB treatment were tested on NBA/PDA (0.5 g/0.5 ml, 25℃-30℃) **(b)** Fungi (*Alternaria solani, Botrytis cinerea, Colletotrichum gloeosporioides, Fusarium solani, Phytophthora drechsleri, Pythium aphanidermatum,* and *Sclerotinia sclerotiorum)* inoculated into soil treated with autoclave (120 °C / 20 min.) were irradiated with EB 20 kGy and their sterilization were identified by plating on PDA (50 g/500 ml, 25℃). The survival of bacteria (*Bacillus amyloliquefaciens* and *Pseudomonas toraasii)* after 20 kGy EB was identified by plating on NBA (50 g/500 ml, 30℃)**. (c)** Sterilization of *Cylindrocarpon destructans* was identified by PDA. **(d)** Sterilization of *Cylindrocarpon destructans* was identified by PCNB medium.

Supplementary-1 Figure 9. Real time PCR Ct (cycle threshold) value of Nov. 2011 colonies of *Cylindrocarpon destructans* isolates formed on PCNB agar medium from 2009 Jinan 1st root field soil based on *Cylindrocarpon destructans* colonies using amplification curve analysis (3×10^5/㎖, 3×10^4/㎖, and 3×10^3/㎖, respectively) of SYBR Green I real-time with primer sets CDPCF12/CDPCR121 and CDIGS47NF2/CDIGS47NR1.

Supplementary-1 Figure 10. Ct (Cycle threshold) value standard curve on sensitivity of SYBR Green I PCR assessed by 10-fold serial dilutions of genomic DNA *Cylindrocarpon destructans* (CY2009) extracted from pure cultures with primer set CDPCF12/CDPCR121. **(a)** Real-time amplification curve of different concentration of *Cylindrocarpon destructans* (3×10^5/㎖, 3×10^4/㎖ and 3×10^3/㎖, respectively) of DNA. Line 1: 10-fold dilution of CY2009 DNA (10 ng/ul - 1 pg/ul). **(b)** Melting curve analysis. **(c)** Standard curve analysis of the relationship between copy

number and corresponding Ct value was reported.

Supplementary-1 Figure 11. Phylogenetic analysis on *Cylindrocarpon destructans.*

Supplementary-1 Figure 12. (a) Transparency solar panel sunshine shield concept design for facilitating medicinal root settlement field. **(b)** Overall view of the concept design of a settlement of the ongoing medicinal root field using EB treatment and permanent sunshine shield (or transparency solar panel).

Supplementary-1 Table Legends

Supplementary-1 Table 1. Root survival rate (%).

Supplementary-1 Table 2. Status of root growth (cm, ea, and g).

Supplementary-1 Table 3. Status of root stem growth (cm).

Supplementary-1 Table 4. Disease occurrence rate on root (%).

Supplementary-1 Table 5. The contents of major ginsenosides in root.

Supplementary-1 Table 6. Microorganism density (CFU/g) in soil.

Supplementary-1 Table 7. (a) Soil nutrient mineral analysis after 20 kGy EB treatment.

Supplementary-1 Table 7. (b) Soil monitoring on EB effect on soil physiochemical property of mineral nutrients. Date: June 2009. 3 iteration test (P=0.05).

Supplementary-1 Table 7. (c) Soil monitoring on EB effect on soil physiochemical property of mineral nutrients. Date: June 2010. 3 iteration test (P=0.05).

Supplementary-1 Table 7. (d) Soil monitoring on EB effect on soil physiochemical property of mineral nutrients. Date: June 2011. 3 iteration test (P=0.05).

Supplementary-1 Table 8. Neutron activation analysis on root soil irradiated by Electron beam, 3 iteration test.

Supplementary-1 Notes

Supplementary-1 Note 1. Data Sheet of electron beam treatment throughput calculation on cultivation soil [1].

Supplementary-1 Note 2. Economic efficiency evaluation for ongoing settlement cultivation field of eco-friendly medicinal root plant (*Panax ginseng)*[2-4].

Supplementary-1 Movies (Recorded on 1 June 2012)

Supplementary-1 Movie 1. Jinan root field (2008-2012); recorded on 1 June 2012.

Supplementary-1 Movie 2. 5-year-old root field with first planting soil (FS); recorded on 1 June 2012.

Supplementary-1 Movie 3. 5-year-old root field with replanting soil (RS) non-treated with EB: Harvest 6-year-old root on Nov. 2008 and transplant root seedlings on Mar.2009; recorded on 1 June 2012.

Supplementary-1 Movie 4. 5-year-old root field with replanting soil (RS) treated with electron beam 20 kGy: Harvest 6-year-old root on Nov 2008, soil management with EB 20 kGy on Feb. 2009, and transplant root seedlings on Mar. 2009; recorded on 1 June 2012.

Supplementary-1 Movie 5. 4-year-old root field with first planting soil (FS); recorded on 1 June 2012.

Supplementary-1 Movie 6. 4-year-old root field with replanting soil (RS) non-treated with EB: Harvest 6-year-old root on Nov. 2008, soil management with planting Sudan grass on May 2009, and transplant root seedlings on Apr.2010; recorded on 1 June 2012.

Supplementary-1 Movie 7. 4-year-old root field with replanting soil (RS) treated with electron beam 20 kGy: Harvest 6-year-old root on Nov. 2008, soil management with planting Sudan grass on May 2009, soil management with EB 20 kGy on Feb. 2009, and transplant root seedlings on Apr.2010; recorded on 1 June 2012.

Supplementary-1 Figures Reference

1. Guide line for testing of chemicals: Earthworm, acute toxicity tests. *Organization for Economic Cooperation and Development* **207** (1984).

2. Technical Recommendation for the update of the ISO Earthworm Field Test Guide line*. **ISO*** **11268-3**(2006) .

3. Song, J.Y., Kim, S.I., Seo, M.W. & Kim, H.G. Analysis of genetic diversity and development of PCR assay for ginseng root rot pathogen, *Cylindrocarpon destructans*. *Korean Society Microbiology Newsletter* **22**, 13-16 (2010).

Supplementary-1 Note Reference

1. Fairand, B.P. Radiation sterilization for health care products: X-ray, gamma, and electron Beam. *CRC PRESS*, 2002.

2. Kimberly, A.G. & Marshall, R. C. Environmental radiolysis for soil and sediment treatment: A review of chemistry, design, and economic issues. *J. Adv. Oxid. Technol* **3-1,** 22-36 (1998).

3. Rejuvenation of Industry ginseng & medical plants. *KRDA-Ginseng & Medical Plants Institute Symposium*, **11-1390762-000001-01** (2007).

4. www.kgc.or.kr

5. Mok, S.K. Research prospect and transition of shading of Korean ginseng field *The Korean Ginseng Research at Industry* **5**, 22-35 (2011).

Supplementary-1 Figures

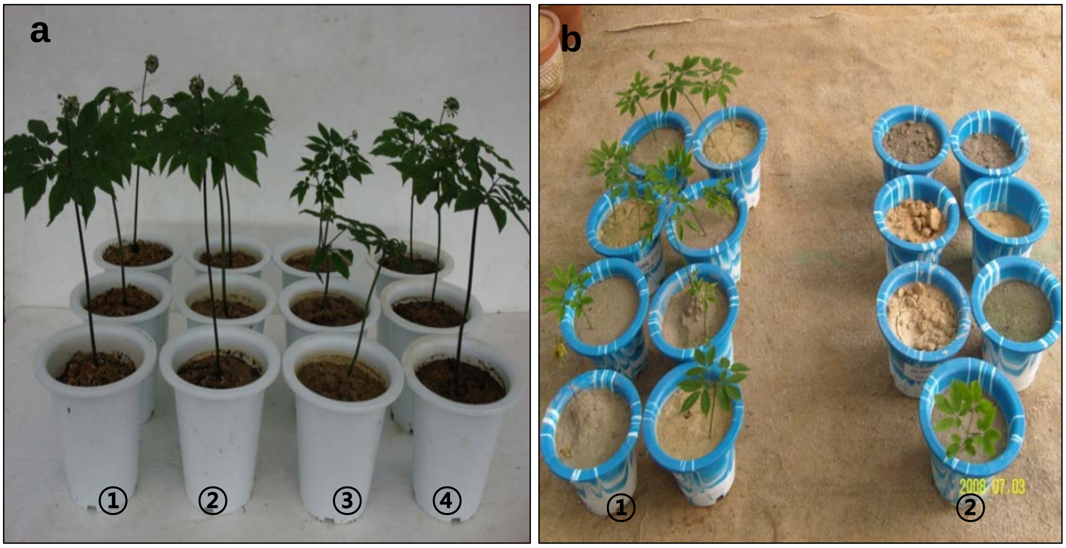

Supplementary-1 Figure 1. 3-4-year-old root planted in pots (2007-2008) for radiation soil treatment experiment. **(a)** One month of growth after gamma irradiation on soil, 5 kGy, May 2007. ① Irradiated first planting soil (FS), ② Irradiated replanting soil (RS), ③ Non-irradiated RS, ④ Non-irradiated FS. **(b)** One month of growth after EB irradiation on soil, 20 kGy, June 2008. ① Irradiated RS, ② Non-irradiated RS.

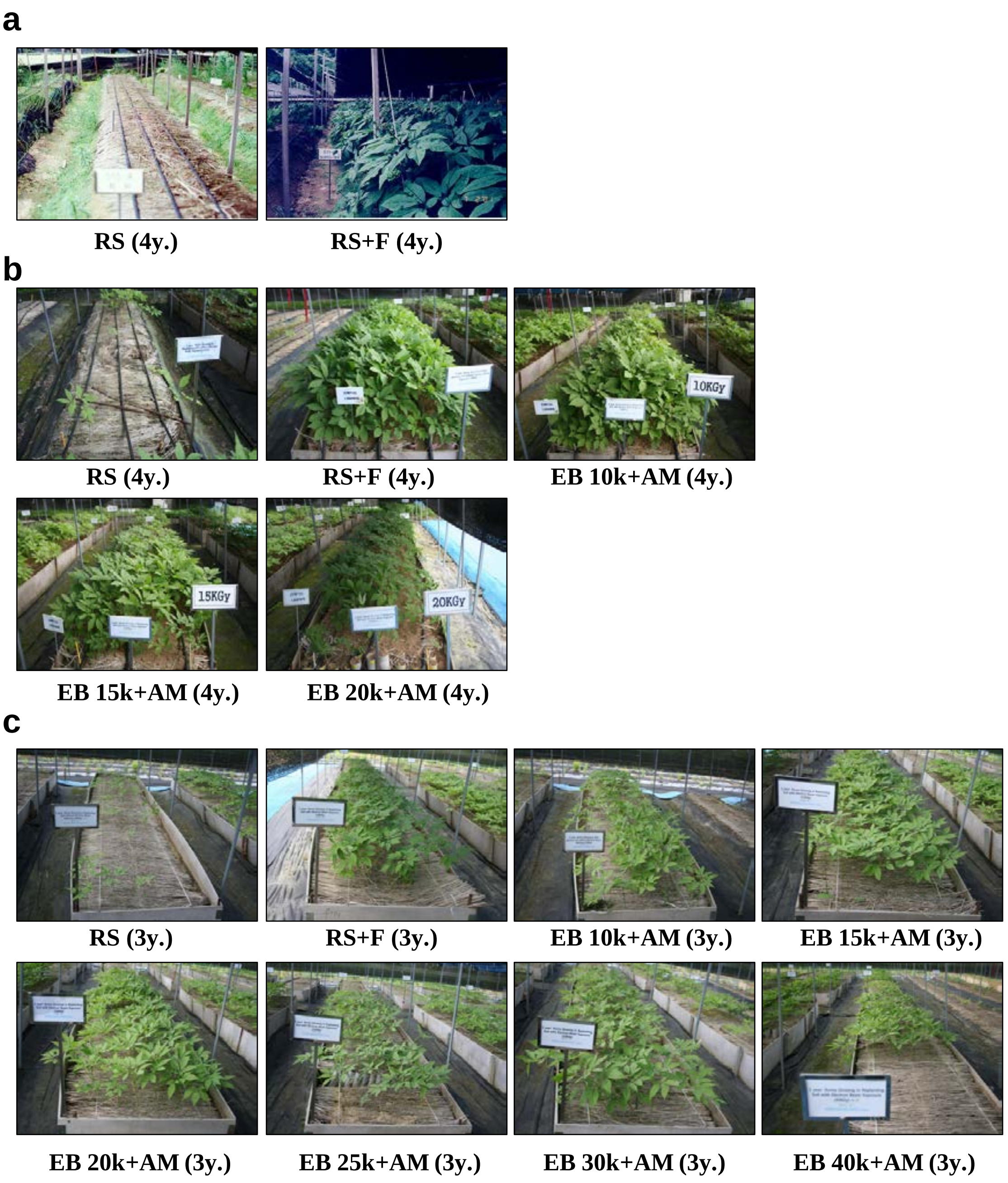

Supplementary-1 Figure 2. Root field (1997-2012) for experiment of treatment of fumigation, electron beam (EB), and antagonistic microorganism (AM). **(a)** Geumsan 4-year-old root(4y.) field (1997-2001). RS (4y.): Replanting soil, RS+F (4y.): RS treated with fumigation (June 2001). **(b)** Jinan 1st 4y. field (2008-2012: Harvested 6-year-old root on 2008, seedling transplant on Apr. 2009, 4y. on June 2011). RS (4y.): Replanting soil, FS(4y.): First planting soil, EB 10k+AM(4y.): Replanting soil treated with EB 10 kGy and AM, EB 15k+AM (4y.): Replanting soil treated with EB 15 kGy and AM, EB 20k+AM (4y.): Replanting soil treated with EB 20 kGy and AM. **(c)** Jinan 2nd 3-year-old root (3y.) field (2008- 2012: Harvested 6- year-old root on 2008, pre-seedling management with rye on 2009, seedling transplant on Apr. 2010, 3y. on June 2011). RS (3y.): Replanting soil, FS(3y.): First planting soil, EB 10k+AM (3y.): RS treated with EB 10 kGy and AM, EB 15k +AM (3y.): RS treated with EB 15 kGy and AM, EB 20k+AM (3y.): RS treated with EB 20 kGy and AM, EB 25k+AM (3y.): RS treated with EB 25 kGy and AM, EB 30k+AM (3y.): RS treated with EB 30 kGy and AM, EB 40k+AM (3y.): RS treated with EB 40 kGy and AM.

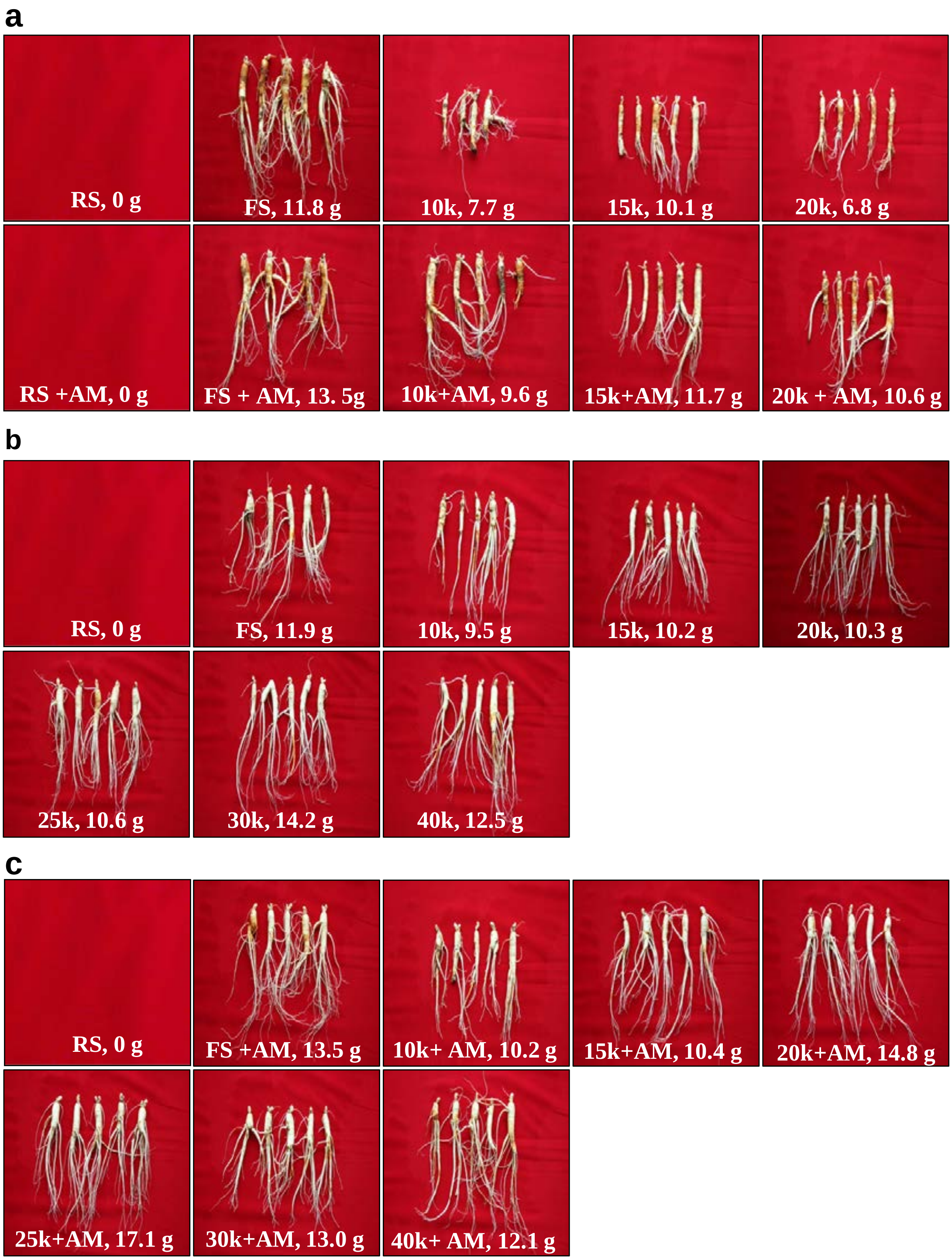

Supplementary-1 Figure 3. 3-4 year-old roots growth in replanting soil (RS), first planting soil (FS), first planting soil treated with AM (FS+AM), 0-40 k: RS treated with electron beam (EB) 10 kGy-40 kGy, and 10-40 k+AM: RS treated with electron beam (EB) 10 kGy-40 kGy and antagonistic microorganism(AM). **(a)** Jinan 1st root field (2009 - 2012: RS soil treated EB on Feb. 2009, transplant root seedlings on Apr. 2009, and harvest 4 year-old roots on Nov. 2011). **(b-c)** Jinan 2nd root field (2008 - 2011: Plant rye for soil management on 2009, RS soil treated with EB on Feb. 2010, transplant root seedlings on Apr. 2010, and harvest 3 year-old root on Nov. 2011).

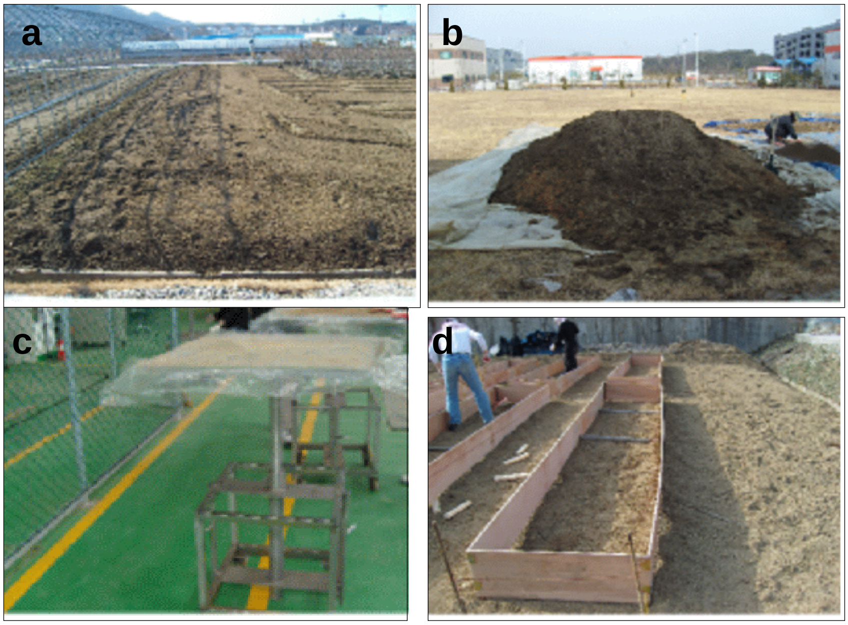

Supplementary-1 Figure 4. The soil (100 tons) from fields in which roots were already grown for 6 years and harvested was carried to the EB facility to be treated by EB. **(a)** Jinan field after roots were grown for 6 years and harvested. **(b)** The pre-used soil carried to the EB facility. **(c)** The pre-used soil conveyed to the EB facility exposure nozzle. **(d)** root field wood frame (1.0 m (W) x 5.0 m (L) x 0.5m (H)) for electron beam experiment.

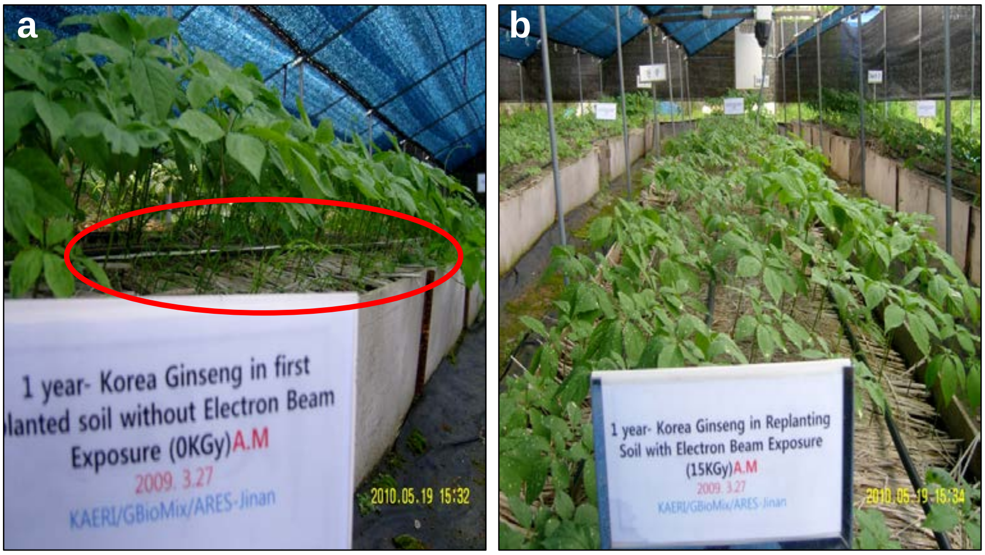

Supplementary-1 Figure 5. The weeds were not grown in irradiated soil. **(a)** Red circle: The weeds of *Stellaria aquatic, Chenopodium album, Alppecurus aequalis* and *Rorippa island* were grown in first planting soil(FS) field. **(b)** The weeds of *Stellaria aquatic, Chenpodium album, Alppecurus aequalis* and *Rorippa island* were not grown in replanting soil(RS) field treated with EB 15 kGy.

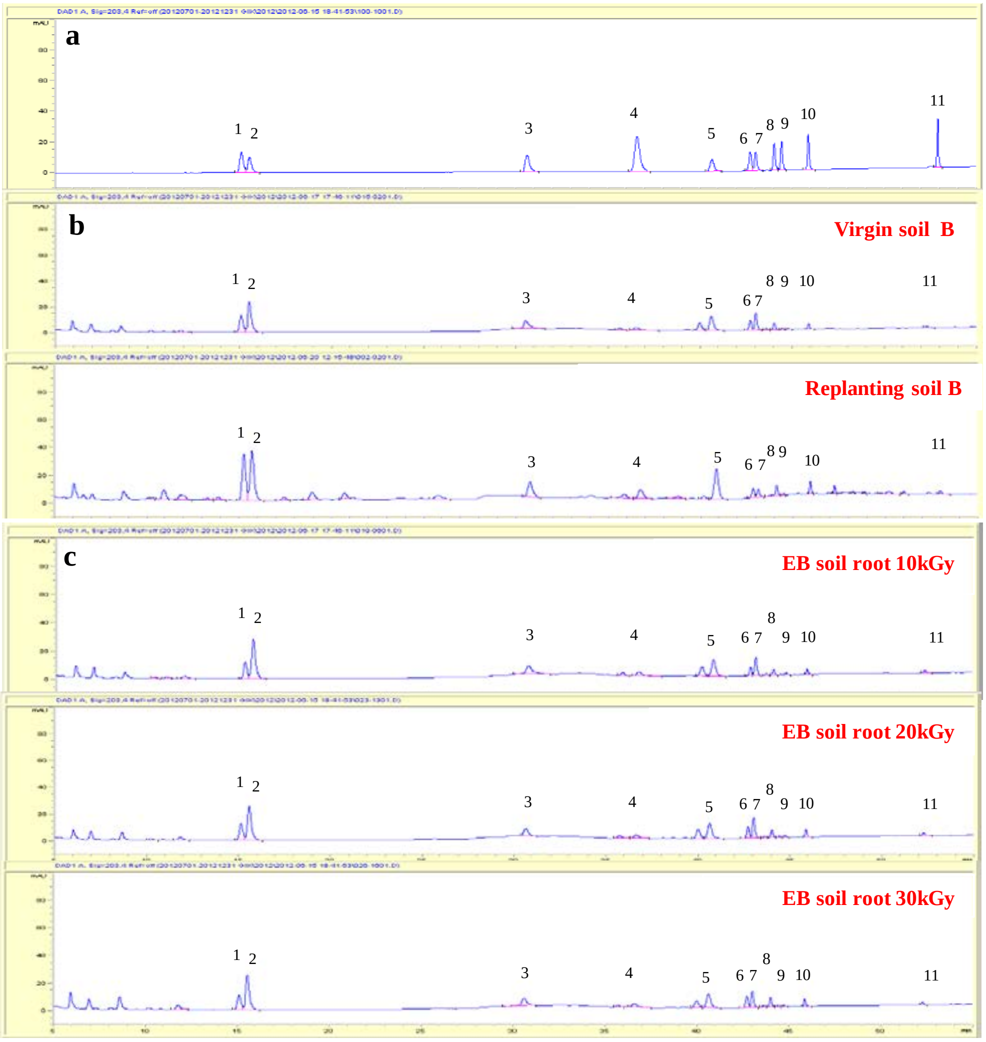

Supplementary-1 Figure 6. HPLC analysis of *root* ginsenosides extracts. **(a)** The major ginsenosides standards of roots. **(b)**The ginsenosides of roots from replanting soil and first planting soil. **(c)** The ginsenosides of roots from EB (0kGy,10kGy, 20kGy, 30kGy) irradiated soil. Ginsenoside standards 1:Rg1, 2:Re, 3:Rf, 4:Rg2+Rh1, 5:Rb1, 6:Rc, 7:Ra1, 8:Rb2, 9:Rb3, 10:Rd, and 11:Rg3

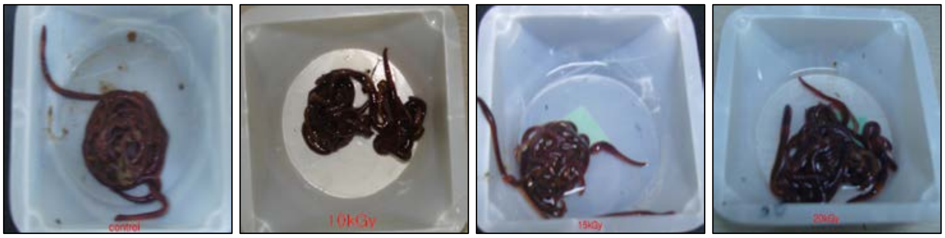

Control **10 kGy** **15 kGy** **20 kGy**

Treatment	Bodyweight of earthworm	
	Before Treatment(g)	Elapsed 30 days(g)
Untreated electron beam soil	1.8	6.0
electron beam 10kGy soil	1.9	6.9
electron beam 15KGy soil	1.7	5.1
electron beam 20kGy soil	1.7	6.1

Supplementary-1 Figure 7. A earthworm field test on EB experiment and its control [1-2]

a

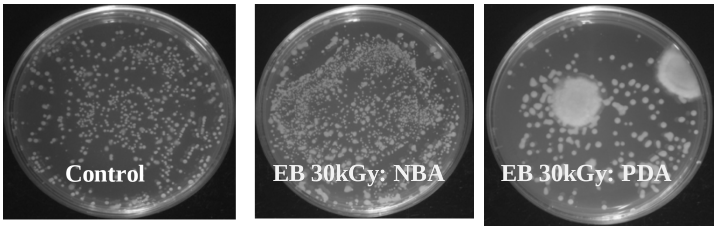

b

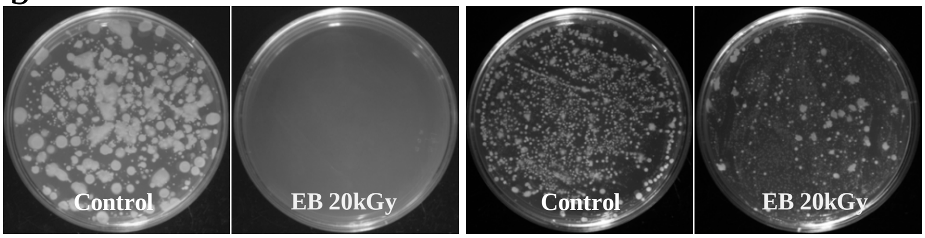

PDA : Fungi after EB 20kGy. **NBA : Bacteria after EB 20 kGy**

c

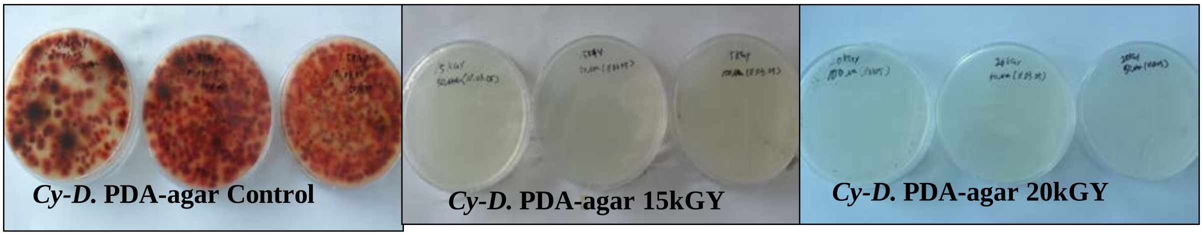

d

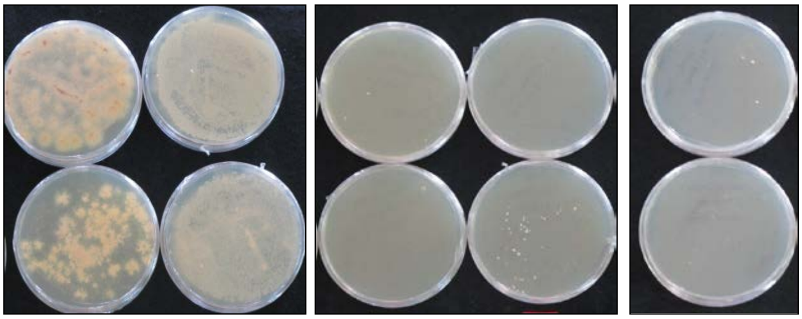

PCNB medium:
distilled water : 1L
peptone: 15g
KH_2PO_4: 1g
$Mg_2SO_4 . 7H_2O$: 0.5g
Agar: 20g
PCBN: 1g
Streptomycin: 0.3g

PCNB Control: *Cy-D.* **PCNB 10-15kGy: *Cy-D.*** **PCNB -20kGy: *Cy-D.***

Supplementary-1 Figure 8. Sterilization of *Cylindrocarpon destrucants*, Fungi, *Actinomyces,* and bacteria with 10 kGy-30 kGy EB were determined by plating on PDA, NBA, and PCNB-agar. **(a)** The viability of *Actinomyces* and bacteria existed in used soil (for 6 years) with EB treatment were tested on NBA/PDA (0.5g/0.5 ml 25°C-30°C) **(b)** Fungi (*Alternaria solani, Botrytis cinerea, Colletotrichum gloeosporioides, Fusarium solani, Phytophthora drechsleri, Pythium aphanidermatum,* and *Sclerotinia sclerotiorum)* inoculated into soil treated with autoclave (120 °C / 20 min.) were irradiated with EB 20 kGy and their sterilization were identified by plating on PDA (50 g/500 ml 25°C). The survival of bacteria (*Bacillus amyloliquefaciens* and *Pseudomonas toraasii)* after 20 kGy EB was identified by plating on NBA (50 g/500 ml 25°C**. (c)** Sterilization of *Cylindrocarpon destructans* was identified by PDA. **(d)** Sterilization of *Cylindrocarpon destructans* was identified by PCNB medium.

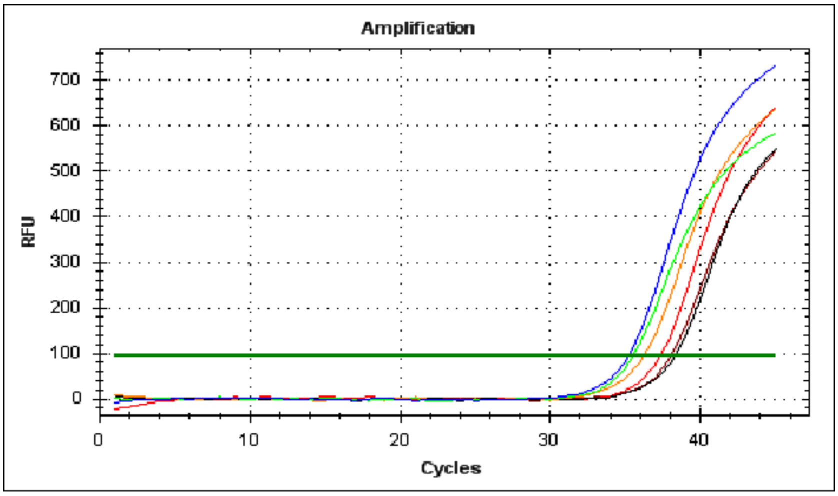

3rd year(2009)	Ct value	Ct value(standard)
FS+AM	37.36	:*C. destructans* 3×10^5/㎖: 27.22<br>:*C. destructans* 3×10^4/㎖: 31.21<br>:*C. destructans* 3×10^3/㎖: 34.90
RS	35.26	
RS+AM	36.26	
RS+AM	35.53	
RS+EB 10kGy	38.08	
RS+EB 15kGy	38.33	
RS+EB 20kGy	No value	

Supplementary-1 Figure 9. Real time PCR Ct(cycle threshold) value of Nov. 2011 colonies of *Cylindrocarpon destructans* isolates formed on PCNB agar medium from 2009 Jinan 1st root field soil based on *Cylindrocarpon destructans* colonies using amplification curve analysis (3×10^5/㎖, 3×10^4/㎖ and 3×10^3/㎖, respectively) of SYBR Green I real-time with primer sets CDPCF12/CDPCR121 and CDIGS47NF2/CDIGS47NR1[3]. FS+AM: First planting soil with treatment antagonistic microorganism, RS: Replanting soil, RS+AM: Replanting soil with treatment antagonistic microorganism, RS+EB (10-20 kGy): Replanting soil treated with electron beam (10-20 kGy),

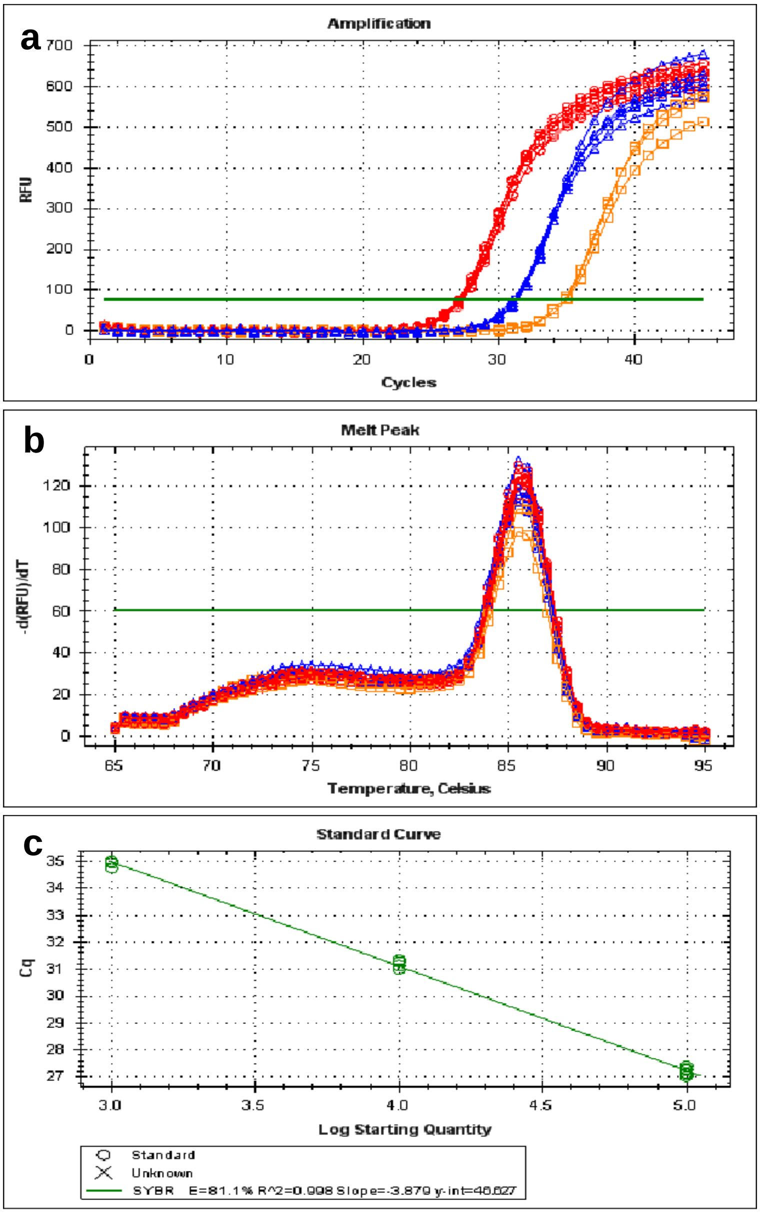

Supplementary-1 Figure 10. Ct (Cycle threshold) value standard curve on sensitivity of SYBR Green I PCR assessed by 10-fold serial dilutions of genomic DNA *Cylindrocarpon destructans* (CY2009) extracted from pure cultures with primer set CDPCF12/CDPCR121. **(a)** Real-time amplification curve of different concentrationof of *Cylindrocarpon destructans* (3×10^5/mℓ, 3×10^4/mℓ and 3×10^3/mℓ, respectively) of DNA. Line 1–: 10-fold dilution of CY2009 DNA (10 ng/ul - 1 pg/ul). **(b)** Melting curve analysis. **(c)** Standard curve analysis. The relationship between copy number and corresponding Ct(Cq) value was reported.

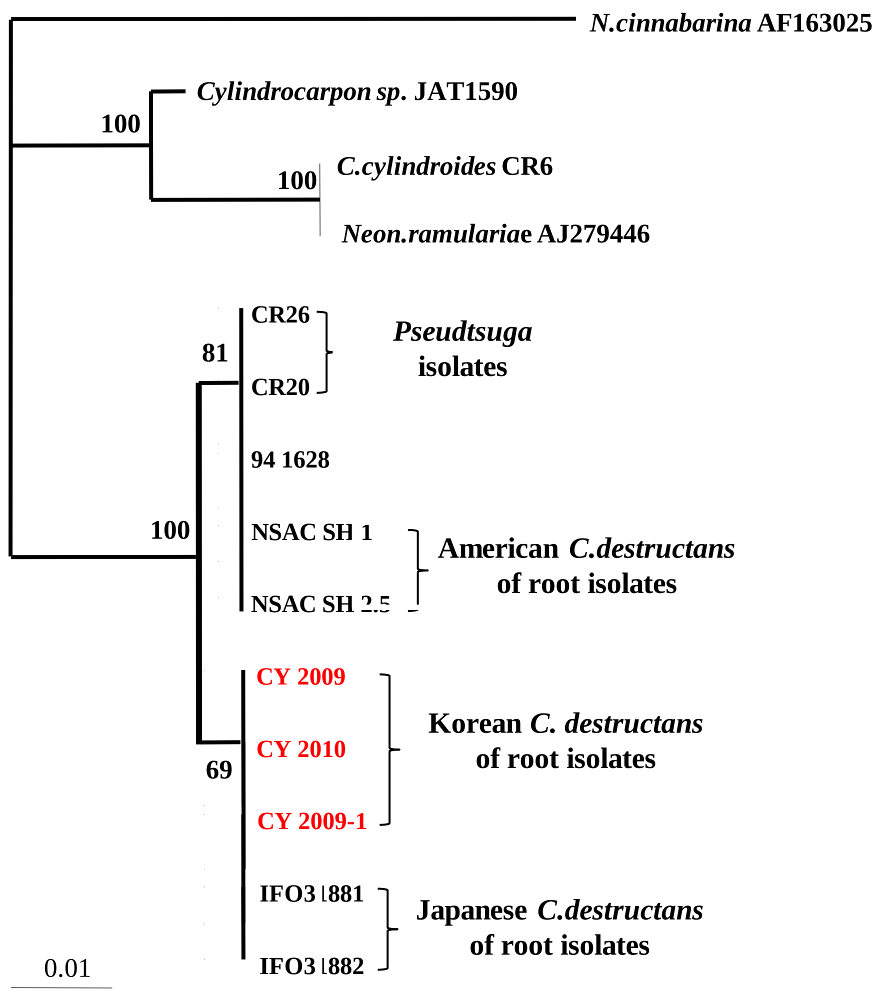

Supplementary-1 Figure 11. Phylogenetic analysis on *Cylindrocarpon destructans.*

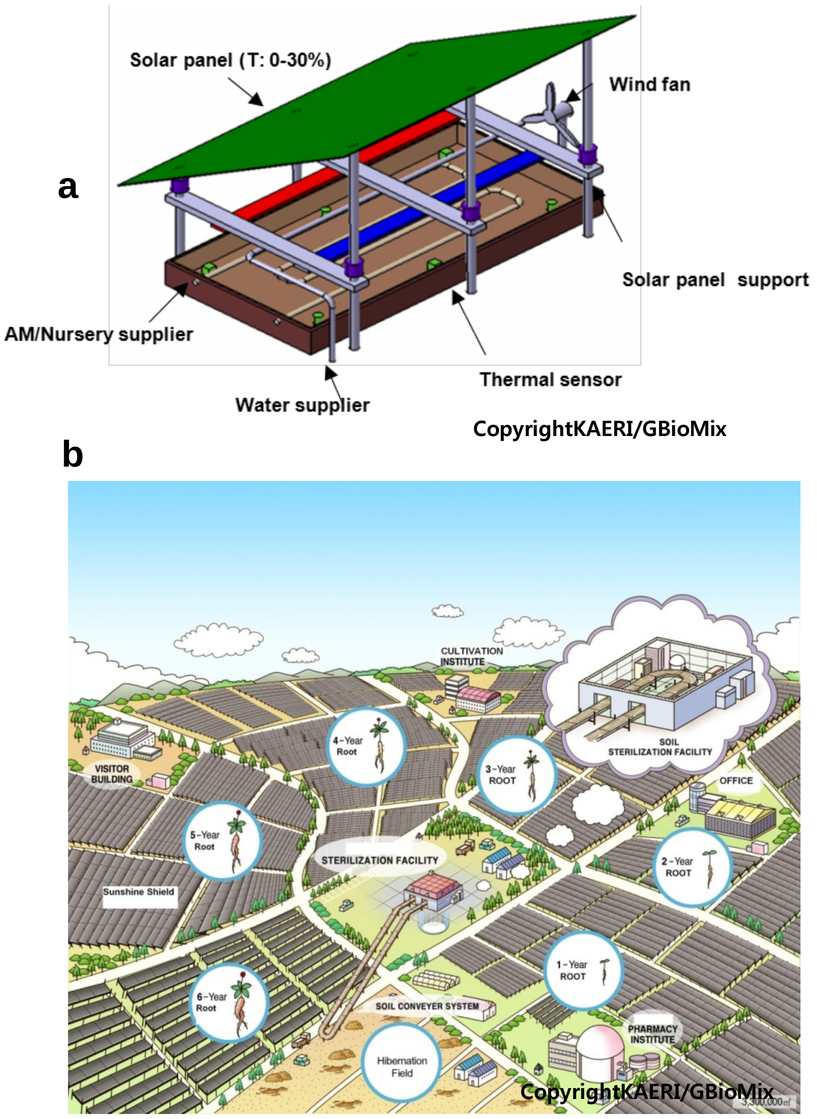

Supplementary-1 Figure 12. (a) Transparency solar panel sunshine shield concept design for facilitating medicinal root settlement field. **(b)** Overall view of the concept design of a settlement of the ongoing medicinal root field using EB treatment and permanent sunshine shield (or transparency solar panel).

Supplementary-1 Figures References

1. Guide line for testing of chemicals: Earthworm, acute toxicity tests .*Organization for Economic Cooperation and Development* **207** (1984).
2. Technical Recommendation for the update of the ISO Earthworm Field Test Guide line. *ISO* **11268-3** (2006).
3. Song, J.Y., Kim, S.I., Seo, M.W. & Kim, H.G. Analysis of genetic diversity and development of PCR assay for root root rot pathogen, *Cylindrocarpon destructans*. *Korea Society Microbiology Newsletter* **22**, 13-16 (2010).

Supplementary-1 Tables

Supplementary-1 Table 1. Root survival rate (%)

ANOVA was carried out using the Costat. Duncan's multiple range test was employed to test for significant differences between the treatments at p=0.05

Root Field	Soil Treatment Condition	1st yr-2nd yr. old root		2nd yr-3rd yr. old root		3rd yr-4th yr. old root		Remark
		N/A	AM	N/A	AM	N/A	AM	
GEUMSAN	RS+IF	96.1 ^{bcd}		11.0 ^{l}		0 ^{i}		- Oct.1997: Harvest 6-year-old root<br>- 1998: Soil management with F&G<br>- Apr.1999: Transplant 1440 root seedlings(GS)<br>- Jun. 1999,2000 and 2001: Survey
	RS+R	95.6 ^{cdef}		37.0 ^{k}		0 ^{i}		
	RS+M	94.6 ^{efg}		1.0 ^{n}		0 ^{i}		
	RS+F	99.0 ^{a}		84.3 ^{f}		56.6 ^{h}		
	RS+F+R	99.1 ^{a}		91.0 ^{b}		70.6 ^{f}		
	RS+F+M	97.1 ^{bc}		90.3 ^{bc}		60.4 ^{g}		
JINAN' -1ST	FS	90.3 ^{i}	92.0 ^{a}	81.0 ^{g}	85.0 ^{d}	76.0 ^{de}	76.1 ^{f}	- Nov.2008: Harvest 6-year-old root<br>- Feb.2009: Soil Management with EB<br>- Mar.2009: Transplant 2492 GS<br>- Jun. 2009, 2010, 2011, and 2012: Survey
	RS	89.0 ^{i}	89.9 ^{a}	5.0 ^{m}	10.0 ^{g}	0 ^{i}	4.2 ^{d}	
	RS+EB 10kGy	89.9 ^{i}	90.1 ^{a}	73.0 ^{j}	76.0 ^{e}	62.1 ^{g}	71.4 ^{gh}	
	RS+EB 15kGy	90.1 ^{i}	94.0 ^{a}	75.0 ^{i}	84.0 ^{d}	61.9 ^{g}	70.9 ^{h}	
	RS+EB 20kGy	94.0 ^{fg}	95.7 ^{a}	89.0 ^{cd}	84.0 ^{d}	77.5 ^{bcd}	79.2 ^{de}	
JINAN -2nd	FS	96.7 ^{bcd}	98.3 ^{a}	81.1 ^{g}	85.7 ^{d}	78.85 ^{ab}	78 ^{ef}	- Nov.2008: Harvest 6-year-old root<br>- May.2009: Soil management with planting rye (Sudan grass)<br>- Feb.2010: Soil management with EB<br>- Apr.2010: Transplant 2856 GS<br>- Jun. 2010, 2011, and 2012: Survey
	RS	93.0 ^{gh}	97.5 ^{a}	12.5 ^{l}	35.9 ^{f}	0 ^{i}	0 ^{j}	
	RS+R+EB 10kGy	98.9 ^{a}	97.4 ^{a}	86.3 ^{e}	88.4 ^{c}	76.8 ^{cde}	80.9 ^{cd}	
	RS+R+EB 15kGy	98.9 ^{ab}	98.9 ^{a}	92.9 ^{a}	94.2 ^{ab}	82 ^{a}	84.2 ^{ab}	
	RS+R+EB 20kGy	97.9 ^{ab}	93.2 ^{a}	88.9 ^{c}	95.7 ^{a}	78.9 ^{bc}	85.7 ^{a}	
	RS+R+EB 25kGy	92.0 ^{h}	92.3 ^{a}	83.8 ^{f}	88.9 ^{c}	74.8 ^{e}	78.8 ^{de}	
	RS+R+EB 30kGy	95.1 ^{def}	96.1 ^{a}	87.6 ^{de}	92.9 ^{b}	79.8 ^{ab}	73.6 ^{g}	
	RS+R+EB 40kGy	92.9 ^{gh}	100 ^{a}	78.6 ^{h}	84.1 ^{d}	78 ^{bcd}	82.9 ^{bc}	
Abbreviation	RS+IF: Replanting soil with IF (Immersion field for 60days), RS+R: Replanting soil mixed with R, RS+M: Replanting soil mixed with M(Manure), RS+F: Replanting soil treated with fumigation, RS+F+R: Replanting soil treated fumigation after R, RS+F+ M: Replanting soil treated with fumigation and manure, FS: First planting soil, RS: Replanting soil, RS+EB (10-20 kGy): Replanting soil treated with electron beam (10-20 kGy), RS+R+EB (10-40 kGy): Replanting soil treated with electron beam (10-40 kGy) after R, AM: treatment, antagonistic microorganism, GS: Ginseng Root Seedlings, NA: No treatment antagonistic microorganism, R: Planting rye (Sudan grass) for pre-seedling soil management.							

Supplementary-1 Table 2. Status of root growth (cm, ea, and g) ANOVA was carried out using the Costat. Duncnas's multiple range to test for significant differences between the treatments at p=0.05

Old	Root growth			Root length		Tap root length		Tap root diameter		Number of fine root		Number of lateral Root		Fresh weight	
	Microorganism			NA	AM	NA	AM	NA	AM	NA	AM	NA	AM	NA	AM
4- yr- old root	GEUMSAN	Soil treat-ment	RS+F	23.4^{b}				15.2^{bc}						20.1^{a}	
			RS+F+R	24.9^{ab}				15.8^{b}						20.3^{a}	
			RS+F+M	25.4^{ab}				14.8^{bc}						19.8^{a}	
	JINAN -1ST		FS	23.7^{b}	26.8^{a}	8.0^{a}	6.8^{a}	18.9^{a}	20.2^{a}	19.8^{a}	16.4^{b}	4.4^{a}	3.6^{b}	11.8^{bc}	13.5^{b}
			RS	0^{f}	0^{f}	0^{b}	0^{b}	0^{e}	0^{e}	0^{g}	0^{g}	0^{g}	0^{g}	0^{g}	0^{g}
			RS+EB 10kGy	11.0^{e}	11.8^{e}	6.1^{a}	6.1^{a}	14.2^{bcd}	15.7^{b}	5.0^{f}	6.6^{ef}	0.8^{f}	1.6^{e}	7.7^{de}	9.6^{cd}
			RS+EB 15kGy	12.7^{e}	17.7^{c}	7.0^{a}	7.2^{a}	14.2^{bcd}	12.8^{cd}	8.2^{de}	13.8^{c}	2.4^{d}	3.4^{bc}	10.1^{cd}	11.7^{bc}
			RS+EB 20kGy	14.7^{d}	18.8^{c}	6.3^{a}	6.3^{a}	11.9^{d}	14.0^{bcd}	8.2^{de}	8.8^{d}	2.8^{cd}	3.2^{bc}	6.8^{e}	10.6^{c}
3-yr- old root	JINAN -2nd		FS	23.2^{d}	26.1^{ab}	7.5^{a}	6.5^{ab}	15.4^{a}	16.3^{ab}	8.4^{e}	19.2^{a}	2.2^{h}	3.8^{d}	11.9^{def}	13.5^{bcd}
			RS	0^{g}	0^{g}	0^{c}	0^{c}	0^{e}	0^{e}	0^{f}	0^{f}	0^{i}	0^{i}	0^{h}	0^{h}
			RS+R+EB 10kGy	19.8^{def}	18.4^{f}	5.2^{b}	7.5^{a}	13.9^{bc}	14.9^{abc}	11.0^{d}	8.0^{e}	2.8^{g}	4.2^{c}	9.5^{g}	10.2^{a}
			RS+R+EB 15kGy	18.5^{f}	24.4^{abc}	6.2^{ab}	6.6^{ab}	15.2^{abc}	15.5^{abc}	11.6^{d}	16.4^{bc}	3.6^{e}	3.4^{a}	10.2^{fg}	10.4^{fg}
			RS+R+EB 20kGy	21.6^{cde}	22.8^{cd}	6.2^{ab}	5.7^{ab}	13.5^{abc}	17.0^{a}	8.4^{e}	15.8^{c}	2.8^{g}	4.2^{c}	10.3^{fg}	14.8^{b}
			RS+R+EB 25kGy	22.4^{cde}	23.2^{bc}	5.8^{ab}	5.3^{b}	13.9^{bc}	10.1^{d}	12.2^{d}	19.2^{a}	3.6^{e}	4.4^{b}	10.6^{efg}	17.1^{a}
			RS+R+EB 30kGy	21.9^{cde}	19.4^{ef}	5.5^{ab}	4.7^{b}	15.8^{abc}	15.9^{abc}	15.2^{c}	18.0^{ab}	4.6^{a}	3.6^{a}	14.2^{bc}	13.0^{cd}
			RS+R+EB 40kGy	23.7^{bc}	26.9^{a}	6.4^{ab}	5.7^{ab}	9.8^{d}	15.1^{abc}	12.4^{d}	11.4^{d}	3.4^{f}	4.2^{c}	12.5^{d}	12.1^{de}
**Abbreviation**			RS+F: Replanting soil treated with fumigation, RS+F+R: Replanting soil treated fumigation after R, RS+F+ M: Replanting soil treated with fumigation and manure, FS: First planting soil, RS: Replanting soil, RS+EB (10-20 kGy): Replanting soil treated with electron beam (10~20 kGy), RS+R+EB (10-40 kGy): Replanting soil treated with electron beam (10-40 kGy) after R, AM: treatment, antagonistic microorganism, GS: Ginseng Root Seedlings, NA: No treatment antagonistic microorganism, R: Planting rye(Sudan grass) for pre-seedling soil management												

Supplementary-1 Table 3. Status of Root stem growth (cm)

ANOVA was carried out using the Costat. Duncan's multiple range test was employed to test for significant differences between the treatments at p=0.05

Old			Plant height		Stem height		Leaf length		Leaf width		Stem diameter		Number of leaf	
			NA	AM	NA	AM	NA	AM	NA	AM	NA	AM	NA	AM
4-yr. old	GEUMSAN	RS+F			30.5^{a}		13.4^{cd}		5.3^{c}					
		RS+F+G			30^{a}		14.3^{abc}		5.2^{c}					
		RS+F+M			27.7^{bc}		13^{d}		5.1^{abc}					
	JINAN -1ST	FS	52.3^{ab}	50.2^{b}	27.7^{bc}	28.7^{b}	13.8^{bcd}	14.7^{ab}	6.0^{ab}	6.3^{a}	5.7^{cd}	6.6^{a}	26.7^{a}	26.0^{b}
		RS	0^{f}	0^{f}	0^{g}	0^{g}	0^{g}	0^{g}	0^{d}	0^{d}	0^{h}	0^{h}	0^{e}	0^{e}
		RS+EB 10kGy	40.7^{e}	51.2^{a}	21.7^{ef}	26.5^{bc}	9.8^{f}	14.8^{a}	4.5^{c}	6.3^{a}	4.2^{fg}	5.9^{ab}	20.7^{d}	25.0^{b}
		RS+EB 15kGy	46^{c}	49.2^{b}	22.7^{de}	26.0^{c}	12.0^{de}	14.3^{abc}	5.5^{abc}	5.8^{abc}	5.0^{de}	5.7^{bc}	22.0^{c}	24.7^{b}
		RS+EB 20kGy	40.2^{e}	43.5^{d}	20.8^{f}	23.5^{d}	11.0^{ef}	12.7^{de}	5.2^{bc}	5.5^{bc}	4.1^{g}	4.7^{ef}	21.0^{d}	21.7^{c}
3-yr. old	JINAN -2^{n}	FS	41.7^{ab}	33.3^{e}	22.7^{bc}	16.7^{b}	12.8^{a}	9.7^{b}	4.7^{a}	4.0^{a}	3.6^{a}	3.3^{a}	20.7^{a}	20.0^{a}
		RS	0^{e}	0^{f}	0^{e}	0^{c}	0^{d}	0^{c}	0^{b}	0^{b}	0^{b}	0^{b}	0^{d}	0^{d}
		RS+R+EB 10kGy	37.2^{d}	45.7^{b}	19.5^{d}	26.5^{a}	10.2^{c}	12.5^{a}	4.5^{a}	5.0^{a}	3.3^{a}	4.2^{a}	17.7^{b}	18.7^{ab}
		RS+R+EB 15kGy	45.7^{a}	47.8^{a}	24.2^{ab}	26.5^{a}	12.0^{ab}	10.3^{b}	4.7^{a}	4.3^{a}	4.2^{a}	3.8^{a}	19.7^{a}	17.0^{c}
	d	RS+R+EB 20kGy	36.3^{cd}	43.3^{c}	22.0^{c}	26.5^{a}	10.7^{bc}	12.2^{a}	4.3^{a}	4.8^{a}	3.4^{a}	3.7^{a}	15.3^{c}	20.3^{a}
		RS+R+EB 25kGy	39.0^{cd}	40.2^{d}	19.8^{d}	26.5^{a}	11.8^{abc}	11.2^{ab}	4.7^{a}	4.7^{a}	3.3^{a}	3.5^{a}	17.0^{b}	18.7^{ab}
		RS+R+EB 30kGy	45.2^{a}	45.5^{b}	24.8^{a}	26.5^{a}	12.8^{a}	12.8^{a}	5.0^{a}	5.0^{a}	3.9^{a}	4.1^{a}	20.0^{a}	17.0^{bc}
		RS+R+EB 40kGy	35.7^{cd}	40.0^{d}	20.3^{d}	26.5^{a}	10.3^{bc}	11.2^{ab}	4.5^{a}	5.0^{a}	3.7^{a}	3.6^{a}	19.7^{a}	20.0^{a}
Abbrev -iation	RS+F: Replanting soil treated with fumigation, RS+F+G: Replanting soil treated fumigation and mixed with grass, RS+F+ M: Replanting soil treated with fumigation and manure, FS: First planting soil, RS: Replanting soil, RS+EB (10-20 kGy): Replanting soil treated with electron beam (10-20 kGy), RS+R+EB (10-40 kGy): Replanting soil treated with electron beam (10-40 kGy) after R, AM: treatment, antagonistic microorganism, NA: No treatment antagonistic microorganism, R: Planting rye (or Sudan grass) for pre-seedling soil management													

Supplementary-1 Table 4. Disease occurrence rate on root (%): 3 iteration test (P=0.05)

Ginseng Field	Plant Disease		Damping off			Anthraenose			Spotting Disease			Phytophthora blight			Root rot		
	Period		1st. year	2nd year	3rd year	1st year	2nd year	3rd year	1st year	2nd year	3rd year	1st year	2nd year	3rd year	1st year	2nd year	3rd year
GEUMSAN	Soil treatment	RS+IF													97.6	100	
		RS+G													43.1	80.	
		RS+M													90.1	100	
		RS+F													7.7	29.3	49.3
		RS+F+G													2.2	18.3	34.2
		RS+F+M													4.2	12.0	44.7
JINAN -1ST		FS	3.3				0.0	1.0	1.0	1.1	5.3<br>**5.1**		0.0	0.0	0	0	0
		RS	9.0				0.0	5.0<br>**3.0**	9.4	0.5	15.0<br>**11.5**		15.7	12.0<br>**10.7**	70	100	100<br>**90**
		RS+EB 10kGy	3.2				0.0	1.0<br>**0.5**	3.8	1.1	2.6<br>**2.1**		0.0	1.0	0	0	1.0<br>**0.0**
		RS+EB 15kGy	4.8				7.4	1.3<br>**1.0**	5.5	4.5	3.0<br>**1.5**		1.1	1.3<br>**10**	0	0	0.0
		RS+EB 20kGy	1.8				1.1	1.0	2.2	1.6	2.3<br>**1.0**		1.5	0.5<br>**0.1**	0	0	0.0
JINAN -2nd		FS				0.0	0.0		0.0	1.0<br>**0.3**		0.0	0.0		0.0	0.0	
		RS				1.0	0.5		1.0	2.0<br>**1.0**		2.5	2.2		60	80<br>**60**	
		RS+R+EB 10kGy				0.0	0.0		0.0	1.0<br>**0.1**		0.0	0.0		0.0	0.0	
		RS+R+EB 15kGy				0.5	0.5		0.5	1.0<br>**0.5**		0.0	0.0		0.5	0.5	
		RS+R+EB 20kGy				0.0	0.6		0.0	0.0		0.0	0.0		0.0	0.0	
		RS+R+EB 25kGy				0.5	0.0		0.5	1.0<br>**0.5**		0.0	0.0		0.5	0.5	
		RS+R+EB 30kGy				0.5	0.5		0.5	1.0<br>**0.5**		0.0	0.0		0.5	0.5	
		RS+R+EB 40kGy				0.0	1.0		0.0	1.0<br>**3.0**		0.0	0.0		0.0	0.0	

Abbreviation: RS+IF: Replanting soil with IF: Immersion Field (60days), RS+R: Replanting soil mixed with M(Manure), RS+F: Replanting soil treated with fumigation, RS+F+R: Replanting soil treated fumigation after R, RS+F+ M: Replanting soil treated with fumigation and manure, FS: First planting soil, RS: Replanting soil, RS+EB (10-20 kGy): Replanting soil treated with electron beam (10-20 kGy), RS+R+EB (10-40 kGy): Replanting soil treated with electron beam (10-40 kGy) after R, AM: treatment, antagonistic microorganism, GS: Ginseng Seedlings, NA: No treatment antagonistic microorganism, R: Planting rye (Sudan grass) for pre-seedling soil management. **Underlined bold**: Antagonistic microorganism treatment.

Supplementary-1 Table 5. The contents of major ginsenosides in roots by cultivation method

ANOVA was carried out using the Costat. Duncan's multiple range test was employed to test for significant differences between the treatments at p=0.05

			Ginsenosides Contents(mg/g)											
**Sample kGy**		**EM**	**Rg1**	**Re**	**Rf**	**Rg2(s)+Rh1(s)**	**Rb1**	**Rc**	**Ra1**	**Rb2**	**Rb3**	**Rd**	**Rg3(s)**	**sum**
**JINAN-1st**	**FS**	T	4.361^{c}	7.902^{a}	1.908^{a}	0.377^{a}	7.667^{a}	2.162^{a}	1.172^{a}	1.797^{a}	0.303^{a}	0.795^{a}	0.000	28.444^{a}
	**FS**	N.T	3.598^{c}	7.824^{ab}	1.882^{a}	0.327^{a}	5.458^{bc}	1.267^{a}	0.954^{a}	1.030^{b}	0.183^{b}	0.328^{d}	0.000	22.852^{c}
	**FS**	N.T	5.223^{bc}	6.485^{ab}	1.767^{a}	0.342^{a}	6.821^{ab}	1.252^{a}	1.077^{a}	1.037^{b}	0.194^{b}	0.535^{b}	0.000	24.732^{b}
	**RS+EB** 10kGy	T.	4.074^{c}	6.464^{ab}	1.702^{a}	0.319^{a}	4.762^{c}	1.057^{a}	0.718^{a}	1.011^{b}	0.167^{b}	0.390^{c}	0.000	20.663^{d}
	**RS+EB** 10kGy	N.T	6.980^{c}	7.552^{ab}	2.405^{a}	0.345^{a}	6.743^{ab}	1.170^{a}	0.596^{a}	1.085^{b}	0.169^{b}	0.460^{b}	0.000	27.504^{a}
	**RS+EB** 20kGy	T.	6.911^{b}	7.236^{ab}	2.130^{a}	0.387^{a}	7.081^{ab}	1.407^{a}	0.870^{a}	1.182^{ab}	0.217^{b}	0.369^{c}	0.000	27.790^{a}
	**RS+EB** 20kGy	N.T	8.860^{a}	5.735^{b}	2.783^{a}	0.329^{a}	6.471^{abc}	1.287^{a}	0.859^{a}	1.048^{b}	0.186^{b}	0.342^{d}	0.000	27.901^{a}
**JINAN-2nd**	FS	T.	2.006^{b}	4.758^{b}	1.202^{a}	0.444^{b}	3.036^{b}	1.258^{a}	2.165^{a}	0.711^{a}	0.131^{a}	0.405^{a}	0.000	16.116^{c}
	FS	T.	5.069^{a}	7.431^{a}	2.273^{a}	1.166^{a}	4.934^{ab}	0.886^{a}	0.845^{a}	1.077^{a}	0.270^{a}	0.816^{a}	0.224	24.993^{a}
	FS	N.T	2.783^{b}	5.342^{b}	1.400^{a}	0.420^{b}	3.291^{b}	1.095^{a}	2.051^{a}	0.610^{a}	0.114^{a}	0.371^{a}	0.000	17.477^{bc}
	FS	N.T	5.642^{a}	5.977^{ab}	1.927^{a}	0.357^{b}	5.528^{a}	1.247^{a}	1.646^{a}	1.236^{a}	0.361^{a}	0.689^{a}	0.000	24.609^{a}
	**RS+EB** 10kGy	T	1.732^{b}	5.565^{ab}	1.119^{a}	0.423^{b}	3.212^{b}	0.942^{a}	2.065^{a}	0.541^{a}	0.000^{a}	0.358^{a}	0.000	15.955^{c}
	**RS+EB** 10kGy	NT	2.208^{b}	5.893^{ab}	1.308^{a}	0.380^{b}	3.514^{b}	1.174^{a}	1.579^{a}	0.788^{a}	0.144^{a}	0.391^{a}	0.000	17.378^{bc}
	**RS+EB** 20kGy	T	1.945^{b}	5.425^{b}	0.980^{a}	0.422^{b}	3.261^{b}	1.373^{a}	2.610^{a}	0.742^{a}	0.138^{a}	0.502^{a}	0.000	17.397^{bc}
	**RS+EB** 20kGy	NT	1.448^{b}	5.132^{b}	0.917^{a}	0.446^{b}	3.003^{b}	1.195^{a}	2.263^{a}	0.674^{a}	0.126^{a}	0.412^{a}	0.000	15.616^{c}
	**RS+EB** 30kGy	T	2.301^{b}	6.078^{ab}	1.445^{a}	0.551^{b}	3.574^{b}	1.266^{a}	2.449^{a}	0.712^{a}	0.136^{a}	0.469^{a}	0.000	18.980^{b}
	**RS+EB** 30kGy	NT	1.938^{b}	5.552^{ab}	1.293^{a}	0.547^{b}	3.438^{b}	1.387^{a}	2.598^{a}	0.761^{a}	0.141^{a}	0.467^{a}	0.000	18.122^{b}
**Abb**	EM: effective Microorganism T: Treated, NT: Not treated, RS: Replanting soil, FS: First planting soil. RS+EB (10-30 kGy): Replanting soil treated with electron beam (10-30 kGy),													

Supplementary-1 Table 6. Microorganism density (CFU/g) in soil

: ANOVA was carried out using the Costat. Duncan's multiple range test was employed to test for significant differences between the treatments at p=0.05

**Ginseng Field**	**Micro organism**	**Filamentos fungi**		**Actinomyces**		**Aerobic bacteria**		**Remark**
	**Antagonistic Microorganism**	**NA**	**AM**	**NA**	**AM**	**NA**	**AM**	
**GEUMSAN**	RS	7.5 x 10^{5}		3.7 x 10^{2}		5.5 x 10^{8}		
	RS+F	1.4 x 10^{3}		3.2 x 10^{1}		3.9 x 10^{8}		1999: Survey after 10 days of fumigation
**JINAN -1ST**	FS	2.0 x $10^{5\,a}$	3.2 x $10^{5\,e}$	8.5 x $10^{6\,c}$	1.7 x $10^{7\,c}$	1.0 x $10^{6\,b}$	1.0 x $10^{6\,c}$	Survey : Mar.2009, Jun.2009, and Sep.2009
	RS	4.7 x $10^{5\,d}$	5.6 x $10^{5\,d}$	7.3 x $10^{6\,c}$	8.0 x $10^{6\,e}$	0.7 x $10^{6\,c}$	1.0 x $10^{6\,c}$	
	RS+EB 10kGy	5.5 x $10^{5\,c}$	6.0 x $10^{5\,c}$	1.0 x $10^{7\,b}$	1.4 x $10^{7\,d}$	0.8 x $10^{6\,c}$	1.8 x $10^{6\,b}$	
	RS+EB 15kGy	6.3 x $10^{5\,b}$	8.0 x $10^{5\,b}$	2.4 x $10^{7\,a}$	2.7 x $10^{7\,b}$	7.5 x $10^{5\,c}$	2.0 x $10^{6\,a}$	
	RS+EB 20kGy	7.5 x $10^{5\,a}$	1.1 x $10^{6\,a}$	2.5 x $10^{7\,a}$	2.9 x $10^{7\,a}$	1.3 x $10^{6\,a}$	1.7 x $10^{6\,b}$	
**JINAN -2nd**	FS	4.5 x $10^{3\,b}$	5.0 x $10^{3\,c}$	1.6 x $10^{5\,b}$	1.9 x $10^{5\,d}$	1.0 x $10^{6\,f}$	1.9 x $10^{6\,d}$	Survey : Apr.2010, Jun.2010, and Sep.2010
	RS	2.8 x $10^{3\,e}$	6.0 x $10^{3\,b}$	1.1 x $10^{4\,e}$	1.9 x $10^{5\,cd}$	1.0x $10^{6\,f}$	1.0 x $10^{6\,f}$	
	RS+R+EB 10kGy	2.3 x $10^{3\,f}$	1.1 x $10^{4\,a}$	2.3 x $10^{4\,e}$	3.2 x $10^{5\,b}$	1.5 x $10^{5\,f}$	1.4 x $10^{6\,e}$	
	RS+R+EB 15kGy	6.5 x $10^{3\,a}$	4.4 x $10^{3\,c}$	1.9 x $10^{4\,e}$	1.7 x $10^{4\,e}$	7.4 x $10^{6\,a}$	2.3 x $10^{6\,b}$	
	RS+R+EB 20kGy	4.0 x $10^{3\,c}$	6.7 x $10^{3\,b}$	1.1 x $10^{5\,c}$	2.1 x $10^{5\,c}$	2.1 x $10^{6\,b}$	1.8 x $10^{5\,g}$	
	RS+R+EB 25kGy	1.3 x $10^{3\,g}$	4.5 x $10^{3\,c}$	6.6 x $10^{4\,d}$	3.6 x $10^{5\,a}$	1.2 x $10^{6\,e}$	5.5 x $10^{6\,a}$	
	RS+R+EB 30kGy	1.2 x $10^{3\,g}$	5.0 x $10^{3\,c}$	1.6 x $10^{5\,b}$	1.9 x $10^{5\,d}$	1.6 x $10^{6\,d}$	1.3 x $10^{6\,e}$	
	RS+R+EB 40kGy	3.2 x $10^{3\,d}$	6.0 x $10^{3\,b}$	1.9 x $10^{5\,a}$	1.9 x $10^{5\,d}$	1.8 x $10^{6\,c}$	2.1 x $10^{6\,c}$	
**Abbreviation**	RS: Replanting soil , RS+F: Replanting soil treated with fumigation, FS: First planting soil, RS: Replanting soil, RS+EB (10-20 kGy): Replanting soil treated with electron beam (10-20 kGy), RS+R+EB (10-40 kGy): Replanting soil treated with electron beam (10-40 kGy) after R, AM: Treatment antagonistic microorganism, NA: No treatment antagonistic microorganism, R: Planting rye (Sudan grass) for pre-seedling soil management.							

Supplementary-1 Table 7. (a) Soil nutrient mineral analysis 3 days later after electron beam irradiation 20kGy, iteration test (P=0.05)

No	Treatment	pH	EC	NO_3 (mg/kg)	P_2O_5 (mg/kg)	K ($cmol^+$/kg)	Mg ($cmol^+$/kg)	Na ($cmol^+$/kg)	Ca ($cmol^+$/kg)	T-N ratio (%)	T-N ratio (%)	CN ratio (%)	OM (g/kg)
1	20 kGy	5.85	0.04	3.645	0.788	0.080	0.838	0.117	1.901	0.086	0.019	0.166	0.33
2	20 kGy	6.20	0.04	4.670	0.751	0.083	0.794	0.118	1.844	0.112	0.024	0.203.	0.41
3	20 kGy	6.21	0.04	3.610	0.540	0.089	0.089	0.115	1.804	0.116	0.021	0.189	0.37
	**Average**	**6.09**	**0.04**	**3.975**	**0.693**	**0.084**	**0.814**	**0.117**	**1.849**	**0.105**	**0.022**	**0.186**	**0.37**
4	0 kGy	6.27	0.06	6.070	0.632	0.080	0.840	0.118	1.793	0.112	0.021	0.192	0.37
5	0 kGy	6.08	0.08	3.910	0.506	0.086	0.811	0.118	1.763	0.118	0.019	0.159	0.32
6	0 kGy	6.23	0.06	3.930	0.669	0.079	0.799	0.116	1.722	0.118	0.015	0.127	0.26
	**Average**	**6.19**	**0.07**	**4.637**	**0.602**	**0.082**	**0.817**	**0.118**	**1.759**	**0.116**	**0.018**	**0.159**	**0.32**

Supplementary-1 Table 7. (b) Soil monitoring on EB effect on soil physiochemical property of mineral nutrients. Date: June 2009. 3 iteration test(P=0.05)

Field	Treatment	pH H_2O(1:5)	EC (dS/m)	OM (g/kg)	P_2O_5 (mg/kg)	Ex Cation(cmol$^+$/kg)			NH_4^+-N (mg/kg)	NO_3^--N (mg/kg)	T-N (%)
						K	Mg	Ca			
	**KRAD Standard**	**5.0-6.5**	**0.5<**	**20-30**	**250-400**	**0.7-1.0**	**2.0-4.0**	**5.0-6.5**		**50<**	
**Jinan 1st Field (2009)**	FS (NA)	6.5	0.22	33.0	310.0	0.53	0.75	1.00		38.0	
	RS (NA)	6.4	0.17	42.0	379.0	0.58	1.05	0.80		82.0	
	RS (AM)										
	RS+ EB 10KGy (NA)	6.8	0.11	28.0	301.0	0.91	0.84	0.80		40.0	
	RS+ EB 10KGy (AM)										
	RS+ EB 15KGy (NA)	6.8	0.12	34.0	280.0	0.85	1.33	0.80		39.0	
	RS+ EB 15KGy (AM)										
	RS+ EB 20KGy (NA)	6.7	0.10	32.0	299.0	0.65	1.07	0.90		24.0	
	RS+ EB 20KGy (AM)										
**Abbreviation**	FS: First planting soil, RS: Replanting soil, RS+EB (10-20 kGy): Replanting soil treated with electron beam (10-20 kGy)<br>AM: treatment antagonistic microorganism. NA: No treatment antagonistic microorganism, .KRAD: Korea Rural Administration Development.										

Supplementary-1 Table 7. (c) Soil monitoring on EB effect on soil physiochemical property of mineral nutrients. Date: June 2010. 3 iteration test(P=0.05)

Field	Treatment	pH H_2O (1:5)	EC (dS/m)	OM (g/kg)	P_2O_5 (mg/kg)	Ex Cation ($cmol^+/kg$)			NH_4^+-N (mg/kg)	NO_3^--N (mg/kg)	T-N (%)
						K	Mg	Ca			
	KRAD Standard	5.0-6.5	0.5<	20-30	250-400	0.7-1.0	2.0-4.0	5.0-6.5		50<	
Jinan 1st Field (2009)	FS (NA)	6.5	0.08	24.6	200.5	0.56	0.67	0.90		3.4	
	RS (NA)	6.5	0.12	34.3	361.0	0.99	1.44	1.10		5.1	
	RS (AM)										
	RS+ EB 10KGy (NA)	6.5	0.10	29.4	110.0	0.78	2.92	8.30		4.8	
	RS+ EB 10KGy (AM)										
	RS+ EB 15KGy (NA)	6.7	0.09	28.8	229.0	0.80	2.24	7.90		4.5	
	RS+ EB 15KGy (AM)										
	RS+ EB 20KGy (NA)	6.4	0.13	28.8	268.0	0.75	1.06	3.50		4.1	
	RS+ EB 20KGy (AM)										
Jinan 2nd Field (2010)	FS(NA)	6.8	0.07	30.8	259.0	0.59	0.03	2.30			
	FS (AM)										
	RS (NA)	6.7	0.10	33.0	291.0	0.25	0.17	2.50		5.1	
	RS (AM)										
	RS+R+ EB 10KGy (NA)	6.6	0.09	21.7	290.0	0.72	0.33	6.00		5.9	
	RS+ R+EB 10KGy (AM)										
	RS +R+ EB 15KGy (NA)	6.5	0.09	33.0	286.0	0.75	0.20	1.00		4.9	
	RS+ R+ EB 15KGy (AM)										
	RS+R+ EB 20KGy (NA)	6.6	0.10	31.4	281.0	0.10	0.02	8.30		5.0	
	RS+ R+ EB 20KGy (AM)										
	RS+ R+ EB 25KGy (NA)	6.5	0.08	41.6	411.0	0.17	0.47	8.20		7.3	
	RS+ R+EB 25KGy (AM)										
	RS+ R+EB 30KGy (NA)	6.7	0.08	14.9	276.0	0.55	0.90	6.70		4.9	
	RS+ R+ EB 40KGy (NA)	6.5	0.10	33.3	295.0	0.15	0.25	8.20		6.1	
Abbreviation	FS: First planting soil, RS: Replanting soil, RS+EB (10-20 kGy): Replanting soil treated with electron beam (10-20 kGy), RS+R+EB (10-40 kGy): Replanting soil treated with electron beam (10-40 kGy) after R, AM: treatment, antagonistic microorganism, NA: No treatment antagonistic microorganism, R: Planting rye (Sudan grass) for pre-seedling soil management, KRAD: Korea Rural Administration Development.										

Supplementary-1 Table 7. (d) Soil monitoring on EB effect on soil physiochemical property of mineral nutrients. Date: June 2011. 3 iteration test(P=0.05)

Field	Treatment	pH H_2O (1:5)	EC (dS/m)	OM (g/kg)	P_2O_5 (mg/kg)	Ex Cation (mol^+/kg)			NH_4^+-N (mg/kg)	NO_3^--N (mg/kg)	T-N (%)
						K	Mg	Ca			
	KRAD Standard	5.0-6.5	0.5<	20-30	250-400	0.7-1.0	2.0-4.0	5.0-6.5		50<	
Jinan 1st Field(2009)	FS (NA)	6.4	0.29	26.6	296.4	0.37	1.47	6.88	7.8	16.2	0.158
	RS (NA)	5.5	0.36	41.5	375.9	0.69	2.40	6.47	9.0	16.8	0.140
	RS (AM)	6.5	0.69	35.3	530.3	1.17	2.85	8.20	10.6	34.4	0.407
	RS+ EB 10KGy (NA)	6.7	0.31	30.2	263.3	0.78	1.93	5.90	7.0	13.2	0.158
	RS+ EB 10KGy (AM)	6.7	0.22	39.7	218.0	0.81	2.56	6.52	9.5	7.6	0.101
	RS+ EB 15KGy (NA)	6.5	0.32	24.2	213.3	0.74	2.60	6.37	8.7	23.0	0.147
	RS+ EB 15KGy (AM)	6.6	0.26	34.7	284.0	0.80	2.00	6.06	9.2	13.4	0.157
	RS+ EB 20KGy (NA)	6.4	0.31	17.5	216.8	0.80	1.79	5.26	10.9	14.8	0.139
	RS+ EB 20KGy (AM)	6.5	0.25	20.7	215.2	0.58	1.76	5.41	10.6	12.9	0.122
Jinan 2nd Field(2010)	FS(NA)	6.8	0.28	19.2	227.5	0.18	1.30	7.20	6.2	12.6	0.151
	FS (AM)	6.7	0.21	14.3	193.8	0.28	1.49	7.30	4.8	16.5	0.126
	RS (NA)	6.8	0.24	20.6	319.6	0.60	1.88	5.68	8.1	10.1	0.190
	RS (AM)	6.8	0.22	34.1	286.2	0.50	2.53	6.75	9.0	10.6	0.182
	RS+ R+ EB 10KGy (NA)	6.6	0.22	17.6	350.2	0.65	2.73	6.16	8.1	17.1	0.160
	RS+ R+ EB 10KGy (AM)	6.6	0.21	33.1	375.3	0.66	2.44	6.35	8.1	18.2	0.178
	RS+ R+ EB 15KGy (NA)	6.5	0.19	25.4	281.3	0.57	1.98	5.77	6.2	7.3	0.181
	RS+ R+ EB 15KGy (AM)	6.5	0.18	22.4	324.6	0.50	2.16	6.28	2.5	4.2	0.137
	RS+ R+ EB 20KGy (NA)	6.6	0.19	24.0	311.3	0.60	1.84	5.50	6.4	4.2	0.164
	RS+ R+EB 20KGy (AM)	6.6	0.19	23.2	327.5	0.73	1.97	5.82	3.4	2.0	0.158
	RS+ R+EB 25KGy (NA)	6.6	0.22	19.6	270.8	0.62	2.10	6.17	2.0	5.6	0.179
	RS+ R+EB 25KGy (AM)	6.8	0.33	29.1	369.5	0.94	2.44	6.81	4.2	10.4	0.197
	RS+ R+EB 30KGy (AM)	6.7	0.21	25.8	306.8	0.61	2.22	6.17	2.0	5.3	0.174
	RS+ R+EB 40KGy (AM)	6.5	0.21	28.5	334.9	0.64	3.00	6.76	2.8	8.4	0.181
**Abbreviation**	FS: First planting soil, RS: Replanting soil, RS+EB (10-20 kGy): Replanting soil treated with electron beam (10-20 kGy), RS+R+EB (10-40 kGy): Replanting soil treated with electron beam (10-40 kGy) after R, AM: treatment, antagonistic microorganism, NA: No treatment antagonistic microorganism, R: Planting rye (Sudan grass) for pre-seedling soil management, KRAD: Korea Rural Administration Development.										

Supplementary-1 Table 8. Neutron activation analysis on ginseng soil irradiated by electron beam(EB), 3 iteration test

value(ppm)

Sample	Al	Ti	V	Mn	As	Br	Co	Cr	Eu
**EB 10kGy-1**	63630	2826	57	774.9	18.25	2.983	9.128	51	1.39
**EB 10kGy-2**	69770	2782	55.67	511.9	18.17	2.514	10.08	49.64	0.8728
**EB10kGy -3**	70730	4092	82.96	688.2	19.83	2.512	10.89	52.19	0.7838
**EB10 kGy-1**	65000	2107	50.64	723	18.45	1.625	8.932	56.62	1.25
**EB15 kGy-2**	75960	3246	62.01	823.9	15.6	4.9	10.96	53.76	0.9809
**EB15 kGy-3**	75250	3133	50.34	545.3	17.28	2.71	10.18	51.83	0.9435
**EB20 kGy-1**	64440	4443	57.2	591.2	17.35	2.44	9.403	49.11	0.4891
**EB20 kGy-2**	65660	3384	55.34	610.9	17.93	1.959	9.19	49.66	0.821
**EB20 kGy-3**	74550	5088	72.83	763.9	19.67	3.81	9.208	57.4	
**Control**	63790	3628	54.1	674.5	14.78	2.937	10.08	50.57	0.6161
**Control**	69260	4182	58.47	690.1	22.38	3.816	9.382	46.83	1.203
**Control**	74730	3282	71.87	791.5	19.63	2.825	11.28	64.22	1.108

Error (%)

Sample	Al	Ti	V	Mn	As	Br	Co	Cr	Eu
**EB10 kGy-1**	0.24	12.7	5.36	1.29	5.5	19.71	11.5	11.68	29.58
**EB10 kGy-2**	0.51	24.64	11.55	3.31	2.89	11.93	5.06	5.81	18.42
**EB10 kGy-3**	0.39	18.25	8.68	3.4	3.86	26.05	8.07	9.86	36.21
**EB15 kGy-1**	0.52	22.59	9.81	2.01	5.61	59.66	11.25	9.87	23.83
**EB15 kGy-2**	0.4	15.31	7.28	1.99	9	25.36	12.06	14.05	43.18
**EB15 kGy-3**	0.57	21.71	11.62	4.52	3.14	11.82	4.83	5.56	16.7
**EB20 kGy-1**	0.3	12.35	9.78	2.66	5.62	43.2	11.54	12.76	75.76
**EB20 kGy-2**	0.3	11.51	6.61	1.99	4.45	40.61	9.01	9.29	32.83
**EB20 kGy-3**	0.44	16.27	10.71	2.61	5.42	28.83	12.18	11.1	
**Control**	0.32	12.03	9.45	2.39	3.54	14.13	8.73	9.93	49.63
**Control**	0.25	10.97	7.29	1.58	2.77	11.56	9.55	10.8	22.65
**Control**	0.43	19.27	9.22	1.68	4.84	19.87	8.87	9.21	70.29

Continually Supplementary-1 Table 8

Sample	Hf	K	La	Na	Fe	Sb	Sc	Yb
**EB10kGy-1**	5.213	29780	45.18	11990	25070	0.9074	8.573	2.567
**EB10kGy-2**	5.45	28650	42.3	11000	25470	0.697	8.618	3.289
**EB10kGy-3**	6.803	23180	41.61	10820	28560	0.7084	9.563	2.762
**EB15kGy-1**	6.766	19090	40.35	10520	24670	0.5636	8.865	2.685
**EB15kGy-2**	7.603	26180	40.57	10290	25140	0.8267		2.319
**EB15kGy-3**	5.243	25260	38.98	9804	25320	0.878	8.429	2.133
**EB20kGy-1**	5.139	27500	46.38	8816	25400	1.092	8.334	2.717
**EB20kGy-2**	6.04	26290	42.19	9352	26210	0.9188	8.762	
**EB20kGy-3**	5.263	22170	40.73	9106	25540	0.6248	8.512	
**Control**	5.349	22500	40.5	9138	24430	0.9909	8.169	3.133
**Control**	5.222	30580	43.25	9613	27700	1.182	9.485	2.892
**Control**	5.903	28170	41.8	10460	27880	1.016	8.714	2.209

Error (%)

Sample	Hf	K	La	Na	Fe	Sb	Sc	Yb
**EB10kGy-1**	8.41	10.71	0.82	0.86	2.51	17.16	0.97	7.64
**EB10kGy-2**	3.71	6.94	0.42	0.56	1.41	10.84	0.47	2.94
**EB10kGy-3**	5.86	13.85	0.8	0.85	1.86	19.81	0.78	6.05
**EB15kGy-1**	6.67	18.92	0.93	1.02	2.38	27.19	0.92	7.08
**EB15kGy-2**	8.21	17.31	1.23	1.11	3.09	28.57		11.02
**EB15kGy-3**	3.92	7.79	0.51	0.67	1.18	9.3	0.51	4.61
**EB20kGy-1**	9.61	12.7	0.9	0.89	2.45	16.26	1.09	7.82
**EB20kGy-2**	5.94	10.84	0.74	0.81	1.8	15.96	0.79	
**EB20kGy-3**	8.42	18.07	1.07	1.1	2.6	31.02	1.12	
**Control**	4.1	11.98	0.74	0.64	2.14	12.81	0.87	5.22
**Control**	5.68	8.06	0.69	0.57	1.91	8.81	0.77	5.82
**Control**	5.69	15.3	0.88	0.85	2.28	13.41	1	9.11

Supplementary-1 Notes

Supplementary-1 Note 1. Data sheet of electron beam soil treatment throughput calculation on cultivation soil [1]

1. **Absorbed Dose vs. Electron Beam Power(1 kGy= 1 kJ/kg)**

   D (kGy) = P*ε / (M / T)

   Where:

   D = average dose in kGy (kJ/kg)

   P = emitted power in kW (kJ/s) → P = MeV x mA

   ε = total efficiency

   T = treatment time in s

   M = mass in kg

2. **Mass Throughput Rate vs. Electron Beam Power**

   M / T = P*ε / D

   Where:

   ε = e*a: ε = fraction of absorbed beam power, e = fraction of emitted power and a = fraction of absorbed power

3. **Mass Throughput Rate (2.5MeV*100kW)**

   M / T = P*ε / D

   P = Beam Power (kW, kJ/s), 100kW

   D = absorbed dose in kGy (kJ/kg), 15kGy, 20kGy, and 25kGy

   ε = E*A (total eff.)= 0.62

   E (beam emitted eff.): 0.95, A (absorbed eff.): 0.65

   M / T = 100 kW*0.62/15 kGy = 4.1 kg/s = 14.9 ton/hr(@15kGy)

   M / T = 100 kW*0.62/20 kGy = 3.1 kg/s = 11.1 ton/hr(@20kGy)

   M / T = 100 kW*0.62/25 kGy = 2.5 kg/s = 8.9 ton/hr(@25kGy)

   -Soil Treatment Throughput (m^3/hr), M/Tρ, (2.5MeV*100kW, ρ=1.5g/cm^2 –soil density)

   = (14.8 ton/ hr) / (1.5ton/m^3) = 9.8 m^3/hr (@15kGy)

   = (11.1 ton/ hr) / (1.5ton/m^3) = 7.4 m^3/hr (@20kGy)

   = (8.9 ton/ hr) / (1.5ton/m^3) = 5.9 m^3/hr (@25kGy)

   -Soil unit area throughput (m^2/hr), Surface soil = 0.3m

   = (9.8 m^3/hr) / (0.3m) = 32.7 m^2/hr (@15kGy)

= (7.4 m^3/hr) / (0.3m) = 24.7 m^2/hr (@20kGy)

= (5.9 m^3/hr) / (0.3m) =19.7 m^2/hr (@25kGy

-Soil unit area throughput (m^2/year), Surface soil = 0.3m

32.7 m^2/hr (@15kGy) x 4800hr/year (70% operation)=156,960 m^2

24.7 m^2/hr (@20kGy) x 4800hr/year (70% operation)=118,560 m^2

19.7 m^2/hr (@25kGy x 4800hr/year (70% operation)=94,560 m^2

4. Solids handling system (@ 2.5MeV*100kW)

V(conveyor velocity, m/min) = Q(m^3/min)/A(m^2)

Q=9.8 m^3/hr(@15kGy), 7.4 m^3/hr(@20kGy) and 5.9 m^3/hr(@25kGy)

A= conveyor width 1.5 m * Soil depth 5.9 mm = 0.0089 m^2 (@2.5MeV)

Velocity = (9.8 m^3/hr*1hr/60min) / (0.0089m^2) = 18.3 m/min (@15kGy)

= (7.4 m^3/hr*1hr/60min) / (0.0089m^2) = 13.8 m/min (@20kGy)

= (5.9 m^3/hr*1hr/60min) / (0.0089m^2) = 11.0 m/min (@25kGy)

:V = 11.0 - 18.3 m/min

Supplementary-1 Note 2. Economic efficiency evaluation for ongoing settlement cultivation field of eco-friendly medicinal root plant [2-4]

Economic efficiency evaluation for ongoing settlement cultivation of eco-friendly medicinal root plant (*Panax ginseng*) was performed by view point of land cost, growing cost, yield amount and its price. The data is to examine the economic feasibility of newly developed soil remediation techniques to allow settled ongoing cultivation of organic medicinal root plant (*Panax ginseng)*. In according to Fig.3, when cultivation area goes beyond 15,000 m^2, the economic valuation point is feasible. Cultivation scale beyond valuation point will have an investment value.

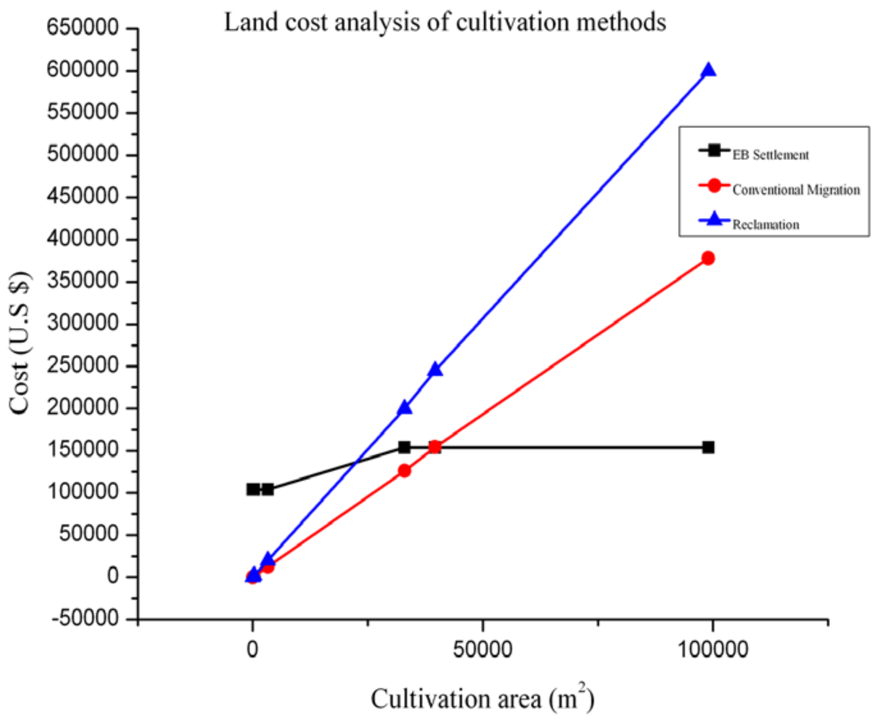

Supplementary-1 Note-2 Figure 1. Land cost analysis for ongoing settlement cultivation of eco- friendly medicinal root plant (*Panax ginseng)* taking account of soil sterilization, soil supply device, soil conveyer device, operation and land rent fee(1.0 US \$/m^2·year).

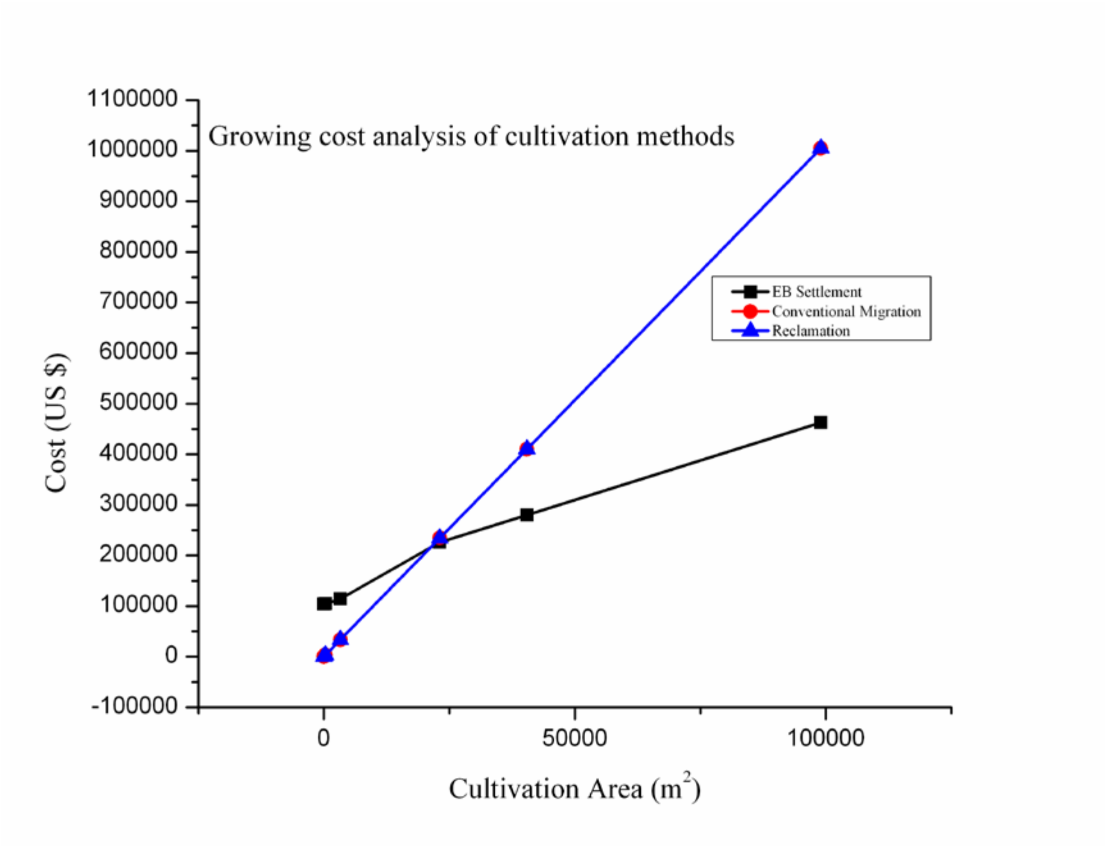

Supplementary-1 Note-2 Figure 2. Growing cost analysis for ongoing settlement cultivation of eco-friendly medicinal root plant (*Panax ginseng)* taking account of soil sterilization, soil supply device, soil conveyer device, operation shading house (20.0 US \$/ m^2) and disease prevention (1.0 US \$/ m^2 ·year).

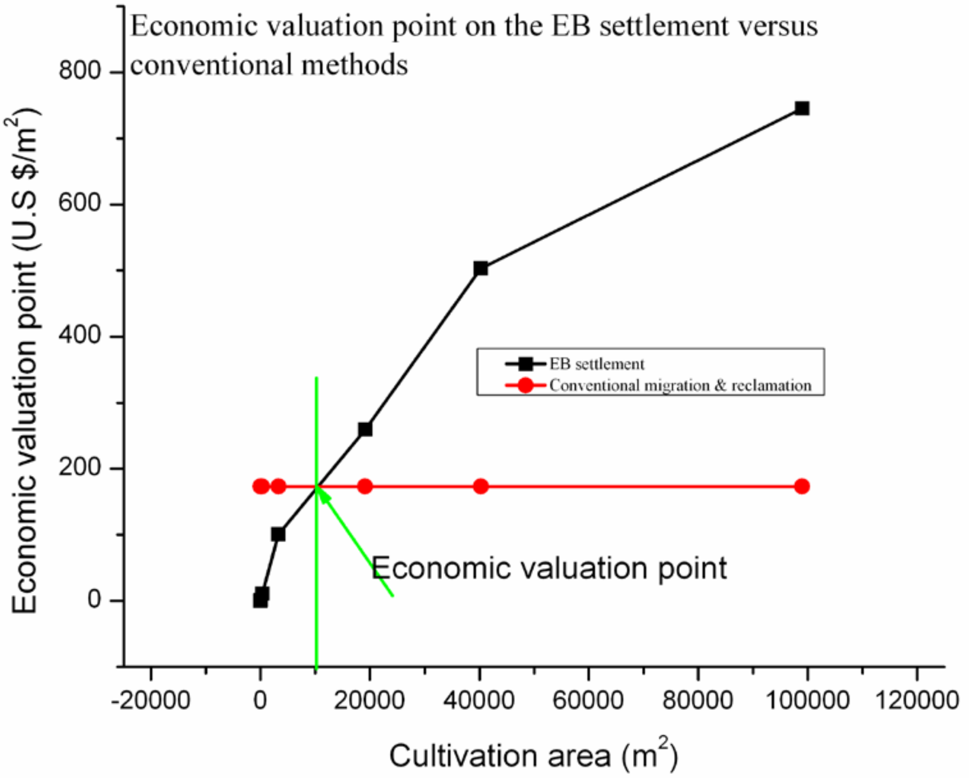

Supplementary-1 Note-2 Figure 3. Economic valuation point analysis for settlement ongoing cultivation of eco-friendly medicinal root plant (*Panax ginseng)* versus conventional methods taking account of the production amount (2.3 kg/m^2) and the price of ginseng. The price of organic ginseng that produced by eco-friendly repeated cultivation is 1.5~2.0 times higher than those by conventional cultivation.

Supplementary Note References

1. Fairand B. P. Radiation sterilization for health care products: X-ray, gamma, and electron Beam. *CRC PRESS*, (2002).
2. Kimberly, A.G. & Marshall, R. C. Environmental radiolysis for soil and sediment treatment: A review of chemistry, design, and economic issues. *J. Adv. Oxid. Technol* **3-1,** 22-36 (1998).
3. Rejuvenation of industry ginseng & medical plants. *KRDA-Ginseng & Medical Plants Institute Symposium,* **11-1390762-000001-01** (2007).

4. www.kgc.or.kr
5. Mok, S.K. Research prospect and transition of shading of korean ginseng field. *The Korean Ginseng Research and Industry* **5,** 22-35 (2011).

Supplementary-2 Information (Neutron Tomography)

Continuous cropping of endangered therapeutic plants *via* electron beam soil-treatment and neutron tomography

Cheul Muu Sim[1, 4], Bong Jae Seong[2], Dong Won Kim[3], Yong Bum Kim[4], Seung Gon Wi[5], Gyuil Kim[6], Hwasuk Oh[1,7], TaeJoo Kim[1], Byung Yeoup Chung[1], Jeong Young Song[8], Hong Gi Kim[8], Sang-Keun Oh[8,9], Young Dol Shin[10,11], Jea Hwan Seok[8,,10], Min Young Kang[10], Yunhee Lee[10,12], Mabuti Jacob Radebe[13], Nikolay Kardjilov[14]& Bernd Honermeier[15]

1. Korea Atomic Energy Research Institute, 1045 Daedeokdaero Yuseong-gu, Daejeon, 303-353, Korea.

2. Geumsan Ginseng & Medicinal Crop Experiment Station, Chungnam, 312-831, Korea

3. Specialized Crop Research Institute, Jinan gun, Jeonbuk, 567-807, Korea

4. National Institute of Horticulture & Herb Science, Bisani 80, Eumseong, Chungbuk, 389-873, Korea

5. Bioenergy Research Institute, Chonnam National University, 300 Yongbong-dong, Buk-gu, Gwangju, 500-757, Korea

6. Institute of Jinan Red Ginseng, Jinan gun, Jeonbuk, 567-801, Korea

7. RIC for Next Generation Industrial Radiation Technology, Wonkwang University. 460, Iksan-daero, Iksan-si, Jeollabuk-do, 54538, Korea

8. Chungnam National University, 220 Gung-dong, Yuseong-gu, Daejeon, 305-764, Korea

9. Plant Genomics and Breeding Institutes, Seoul National University, Gwanak-gu, Seoul 151-921, Korea

10. GBioMix Institute, 723-1, 2 Palbok-dong, Deokjin-gu, Jeonju, 561-844, Korea

11. Chonbuk National University, 567 Baekje-daero, Deokjin-gu, Jeonju, 561-756, Korea

12. King's College London, Palace Road, London, SE1 7EH, U.K

13. Nuclear Energy Corporation South Africa, 0001 Pretoria, South Africa

14. Helmholtz Zentrum Berlin, 14109 Berlin, Germany

15. Justus Liebig University Gießen, Schubertstr. 81, D-35392 Gießen, Germany

Correspondence and requests for materials to C.M.S (cmsim@kaeri.re.kr/cmsimkaeri@msn.com) or B.H (Bernd.Honermeier@agrar.uni-giessen.de)

Supplementary-2 Information Legend

Supplementary-2 Figures

Supplementary-2 Figure 1. Experiment root field with embedded Al pots in soil for diagnosing root pathology by neutron imaging. Al cylinder type pot (diameter of 6 cm and height of 30 cm, and Al pot rectangular type pot (width of 6 cm, length of 20 cm, and height of 30 cm). **(a-c)** Geumsan field (2002-2008); Transplant 2-3- year-old root to 50 Al pots, embedded in soil. **(d-e)** Jinan greenhouse field (Nov. 2007-Nov.2008); Sowed root seeds into 600 Al pots cylinder type embedded in soil. **(g)** Jinan 1st and 2nd field (2008-2012); Transplant root seedling to 600 Al cylinder type pots embedded in field (red circle).

Supplementary-2 Figure 2. Neutron tomography system for the monitoring of root health and the detecting of root pathological change.

Supplementary-2 Method Legends

Supplementary-2 Method 1. Neutron tomography procedure for diagnosing the pathology of root planted in root field.

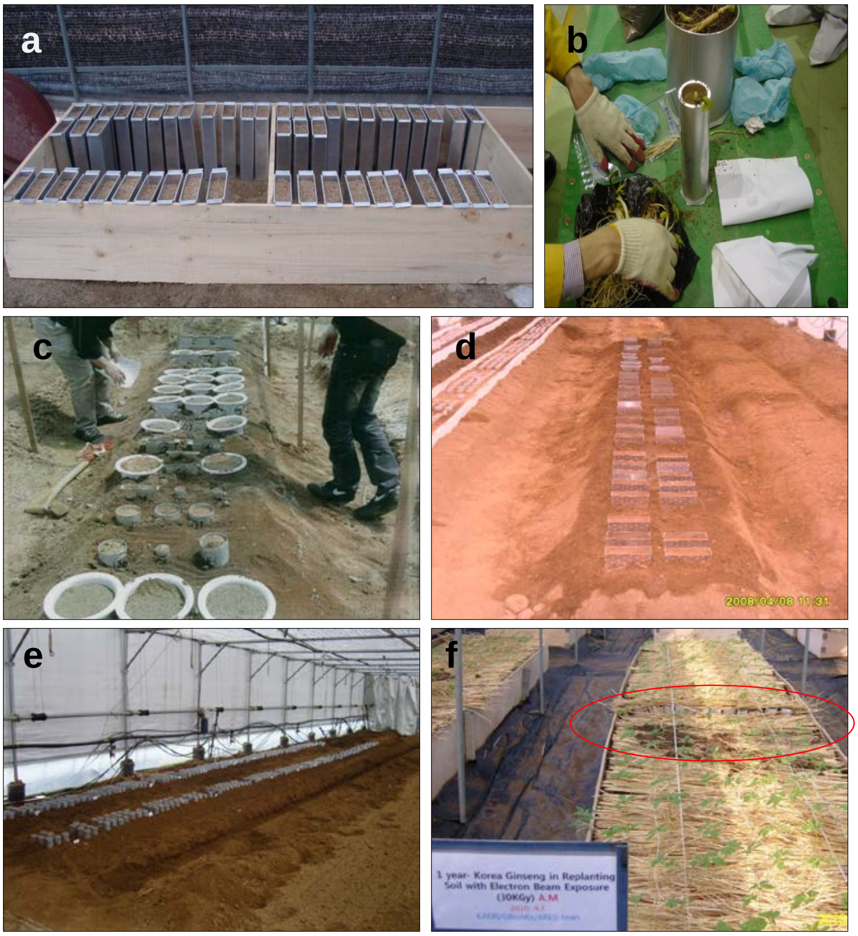

Supplementary-2 Figure 1. Experiment root field with embedded Al pots in soil for diagnosing root pathology by neutron imaging. Al cylinder type pot (diameter of 6 cm and height of 30 cm, and Al pot rectangular type pot (width of 6 cm, length of 20 cm, and height of 30 cm). **(a-c)** Geumsan field (2002-2008): Transplant 2-3- year-old root to 50 Al pots, embedded in soil. **(d-e)** Jinan greenhouse field (Nov. 2007-Nov.2008): Sowed root seeds into 600 Al pots cylinder type embedded in soil. **(g)** Jinan 1st and 2nd field (2008-2012): Transplant root seedling to 600 Al cylinder type pots embedded in field (red circle).

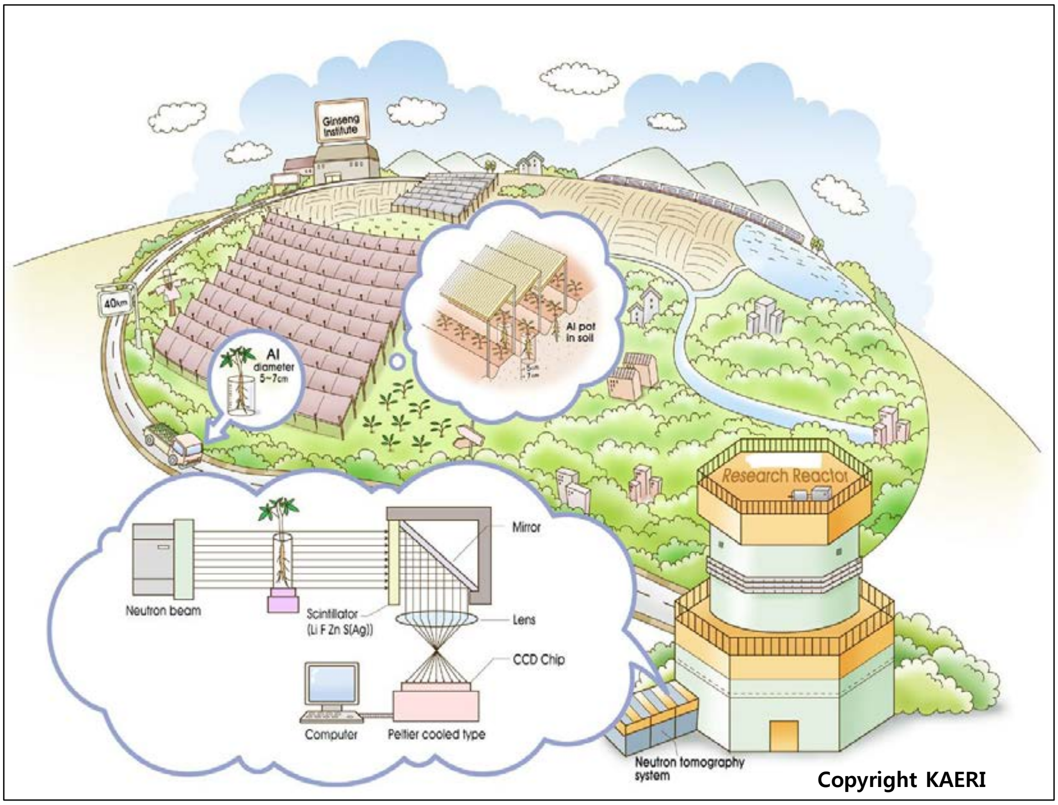

Supplementary-2 Figure 2. Neutron tomography system for the monitoring of root health and the detecting of root pathological change

Supplementary-2 Method

Supplementary-1 Method 1. Neutron tomography procedure for diagnosing the pathology of root planted in the field.

- Al pot (60 mm in diameter, 30 mm in height, 5 mm holes at pot surface and bottom as shown Fig.1) modules made of wood, which is closely mimic to root field's cultural environment are prepared
- Al pot modules made of wood (Fig.2) are buried in root field. Root seedlings and ginseng roots are planted.
- As soon as its withering period ceases, Al pot modules are brought to neutron source. Avoid rain and freezing temperature.
- Only moisture soil existing in Al pots are dried using vacuum drying method (range: 133 Pa.) less than 1 hour
- Tomography scan on Al pots is performed for diagnosing the roots.
- As soon as the scan is completed, Al pot modules are watered.
- Al pot modules are brought back to the field and buried at same place for next tomography scan.

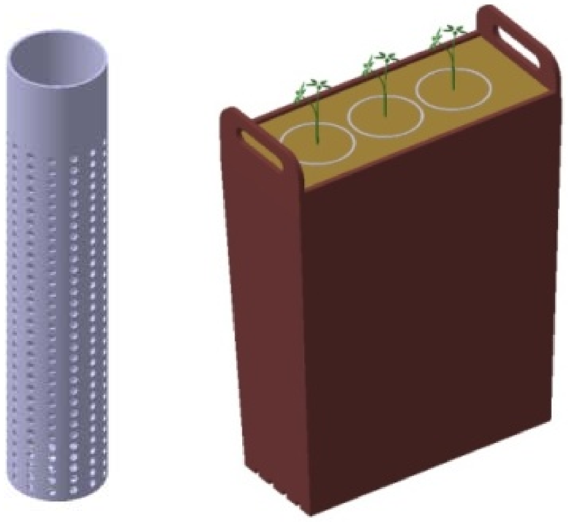

Supplementary Method 1 Figure 1. Al pot and its module made of wood: (30cm(H) x 30cm(L) x 15cm(W))

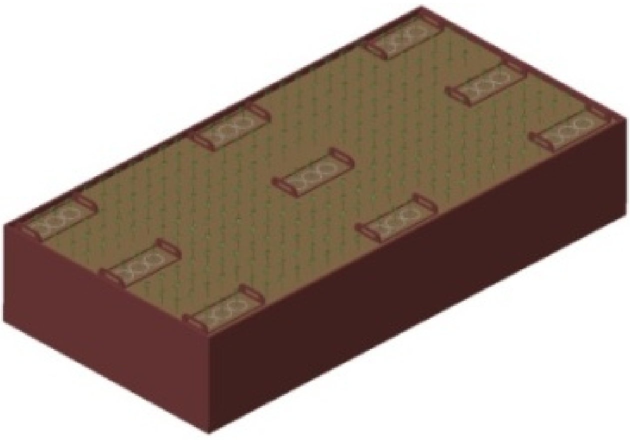

Supplementary Method 1 Figure 2. Root plant field (30cm (H) x 500 cm (L) x 90cm(W))
